# Ongoing structural changes highlight the dynamic nature of chloroplast genomes

**DOI:** 10.1093/nar/gkag117

**Published:** 2026-02-24

**Authors:** Eranga Pawani Witharana, Nobuhiro Kotoda, Kiyohiko Seki, Shinji Fukuda, Yukio Nagano

**Affiliations:** The United Graduate School of Agricultural Sciences, Kagoshima University, Kagoshima 890-0065, Japan; Faculty of Agriculture, Saga University, Saga 840-8502, Japan; The United Graduate School of Agricultural Sciences, Kagoshima University, Kagoshima 890-0065, Japan; Faculty of Agriculture, Saga University, Saga 840-8502, Japan; Faculty of Agriculture, Saga University, Saga 840-8502, Japan; The United Graduate School of Agricultural Sciences, Kagoshima University, Kagoshima 890-0065, Japan; Center for Bioresource Education and Research, Saga University, Saga 849-0903, Japan; The United Graduate School of Agricultural Sciences, Kagoshima University, Kagoshima 890-0065, Japan; Faculty of Agriculture, Saga University, Saga 840-8502, Japan; Vishnu Institute of Pharmaceutical Education and Research, Narsapur 502313, India; Shri Vishnu College of Pharmacy, Bhimavaram 534202, India

## Abstract

High-throughput sequencing has transformed chloroplast genome research by enabling cost-effective analyses from shallow whole-genome sequencing datasets generated using short-read platforms, yet standardized approaches often overlook hidden structural signals critical for understanding plastome evolution. To uncover such signals, we reanalyzed data from our previous study of the subfamily Aurantioideae, which includes *Citrus* and its relatives, and developed a rapid bioinformatic strategy to detect subtle but significant structural rearrangements. Our analysis revealed the emergence of a short inverted repeat pair, which initially destabilizes the plastome and subsequently serves as a substrate for intramolecular homologous recombination. This recombination generates a ∼22 kb inversion in the large single-copy region, producing two distinct genomic configurations. It uncovers structural heteroplasmy across multiple species, which has been maintained since the early Miocene (12.1–28.2 Ma), with the minor isoform becoming dominant in certain lineages through genetic drift. Such structural changes occur gradually rather than as discrete events, representing intermediate stages of plastome evolution. Detection of these stages highlights the dynamic and unstable nature of chloroplast genomes, uncovering a previously underappreciated mechanism generating plastome diversity. Overall, these findings demonstrate that chloroplast genomes are more fluid than previously recognized, with structural transitions actively driving plastome evolution and diversification.

## Graphical Abstract

**Graphical Abstract:**
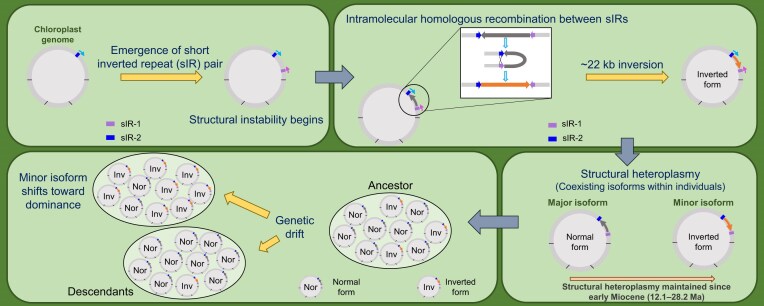

## Introduction

Chloroplast (cp) genomes have gained significant momentum in the scientific community as an emerging focus in plant biology, evolutionary studies, phylogenetics, and genetic diversity, largely driven by the rapid advancement of high-throughput sequencing technologies [1–4]. These technologies have enabled the efficient, accurate, and cost-effective use of cp genome data across a wide range of studies. The intrinsic features of the cp genome, including its relatively small size, conserved structural organization compared to mitochondrial and nuclear genomes, and typical maternal inheritance, offer distinct advantages for evolutionary and phylogenetic analyses [1, 5, 6]. Moreover, variations within the cp genome serve as a rich source of molecular markers that are widely applied in plant systematics, breeding, and conservation efforts [7, 8].

Analyses of cp genomes have become highly standardized. Typically, shallow whole-genome sequencing (WGS) data (∼2–5 Gb) are generated using short-read platforms, followed by assembly with tools such as GetOrganelle, NOVOPlasty, or SPAdes [9, 10, 11]. Annotation is then performed using software including GeSeq, CPGAVAS2, or DOGMA [9, 10, 12, 13], while RNA editing sites and codon usage frequencies are predicted using PREP-cp and CodonW, respectively [14–16]. Whole-genome alignments are commonly visualized with mVISTA, and IR/LSC/SSC boundaries are represented using OGDRAW and IRSCOPE [12, 16–18]. Microsatellites and tandem repeats are detected with REPuter and MISA [12, 16, 19]. Phylogenetic analyses typically rely on concatenated full-length sequences to construct maximum-likelihood (ML) trees with IQ-TREE or RAxML, or Bayesian inference (BI) trees with MrBayes and BEAST [12, 16, 17, 19]. Selection pressures are assessed using Ka/Ks ratios with PAML or KaKs_Calculator, providing insights into purifying selection [12, 16, 17, 19].

While these standard pipelines are valuable for cp genome analyses, they may overlook important signals hidden within short-read data. To address this limitation, we developed a workflow using Read2Tree [20], leveraging the short-read data originally generated for cp genome analysis of the Aurantioideae subfamily to simultaneously analyze a large set of nuclear genes [21]. In our previous study, we revealed significant phylogenetic incongruence between cp-based and nuclear gene-based trees and successfully identified potential sources of these conflicting signals, including incomplete lineage sorting and introgression [21]. Nevertheless, a more comprehensive re-examination of the cp genome data may uncover previously undetected patterns and offer deeper insights into the evolutionary dynamics and complexity of cp genomes.

The cp genome in land plants is typically conserved in structure, exhibiting a quadripartite circular organization composed of two inverted repeats (IRs) flanking the large single-copy (LSC) region and the small single-copy (SSC) region [1]. Despite this structural conservation, pioneering work by Palmer revealed that cp genomes can exist in two distinct isomeric forms within a single species, differing solely in the orientation of the SSC region [22]. This discovery challenged the assumption of structural rigidity in the plastid genome and opened new avenues for exploring its dynamic nature. Subsequent studies extended this observation beyond angiosperms, demonstrating the presence of SSC-inverted isomers in ferns and even in green algae such as *Chlamydomonas* [23]. These findings collectively suggest that the occurrence of cp genome isomers is not restricted to a few taxa but may instead represent a widespread and evolutionarily conserved feature among photosynthetic eukaryotes [23, 24]. Supporting this, Wang *et al.*, utilizing long-read sequencing data, demonstrated that the reversible inversion of the SSC region, resulting in two cp genome isomers, is a common phenomenon across diverse plant lineages [25]. Mechanistically, this inversion is driven by frequent homologous recombination (HR) events between the two large IR regions, which facilitate the flipping of the SSC orientation [22, 26]. Importantly, this recombination-based structural plasticity of the cp genome is not only of evolutionary significance but also has practical applications, as HR in cp genomes has been widely harnessed in cp genetic engineering and transformation strategies [27, 28].

The role of HR in cp genomes extends well beyond the reversible inversion of the SSC region, playing a key role in driving broader structural rearrangements within the plastome [29–32]. Such structural changes are widespread across land plants and have proven to be evolutionarily informative, reflecting both lineage-specific events and deeper phylogenetic divergences. Numerous studies have reported various types of structural changes in the cp genome, including inversions, gene order rearrangements, insertions, deletions, and base substitutions [24, 29, 30, 33]. Among these, large-scale inversion events are particularly notable, as they can significantly alter the structural organization of the cp genome. For instance, in the Asteraceae family, one of the largest families of flowering plants with over twenty thousand species, three major large-scale inversion events exceeding 20 kb have been reported in the cp genome [34]. Two of these occur within the LSC region, while the third involves the SSC region. The occurrence of such extensive inversions across closely related taxa suggests dynamic shifts in cp genome structure over evolutionary timescales, often leading to the emergence of multiple structural forms. This phenomenon is exemplified in the Pinaceae, where four distinct cp genome forms have been identified among closely related species [31]. These forms differ in their inversion patterns and structural rearrangements, highlighting diverse shifts in genome organization within the family [31]. While intramolecular HR, frequently mediated by repeat sequences at rearrangement boundaries, is thought to underlie these inversions, the mechanisms by which cp genomes transition from multiple structural isoforms to a single prevailing configuration remain poorly understood.

Since HR is involved in both the formation of isoforms and structural changes, plastid genomes that are undergoing structural rearrangements may exhibit isoforms with skewed abundance. Clues to such biased isoform ratios may be hidden within the 2–5 Gb of short-read data typically obtained for cp genome analysis. To investigate this possibility, we reanalyzed the short-read datasets originally produced for our previous study of the subfamily Aurantioideae, which includes *Citrus* and its relatives.

In this study, we focused on characterizing a large-scale inversion event that led to substantial structural rearrangements in the cp genome. Although our earlier preprint briefly noted the presence of this inversion [35], a detailed structural analysis was omitted in the peer-reviewed publication, as the final manuscript was streamlined to focus primarily on phylogenetic incongruence [21]. This inversion is most plausibly explained by short inverted repeat (sIR)-mediated HR. By integrating short-read mapping with structural variant analysis, we establish a cost-effective framework for detecting plastome rearrangements directly from short-read data. In particular, we exploit soft-clipped reads (reads that align only partially to the reference due to structural breakpoints), which are typically discarded in standard mapping pipelines, to precisely localize structural breakpoints and infer low-frequency plastome isomers. This approach further revealed the coexistence of alternative cp genome isoforms within individual species, arising from an sIR-mediated inversion that operates independently of canonical flip-flop recombination involving large IRs. Together, these results provide new insights into the dynamic architecture of cp genomes and highlight the evolutionary significance of structural variation. More broadly, our findings suggest that plastome rearrangements play a critical role in lineage diversification and adaptive capacity in land plants, thereby offering an important foundation for future research in plastome evolution, plant systematics, breeding, and conservation biology.

## Materials and methods

### DNA sequence data collection and processing

The DNA used in this study was derived from the same batch as in our previous study and purified following the same protocol [21]. DNA quality was checked by electrophoresis on 1% agarose gel, and concentrations were measured using a DNA BR Assay Kit (Thermo Fisher Scientific, USA). Sequencing libraries were prepared from total DNA by Novogene (Singapore) using the NEBNext Ultra DNA Library Prep Kit for Illumina (NEB, USA) and sequenced on a NovaSeq 6000 platform (Illumina, USA) to generate 150 bp paired-end reads. Details of all short-read data used in this study are provided in Supplementary Table S1. DNA sequence data for *Citrus sinensis* and *Atalantia racemosa* were obtained from public databases (accession numbers in Supplementary Table S1). All sequence data were preprocessed with FastP (v0.23.2) using default settings [36].

### *De novo* assembly and annotation of cp genomes

Cp genomes were assembled *de novo* using the same approach as in our previous study [21] using GetOrganelle (v1.7.5) [37] with R = 15 and k-mer sizes of 21, 45, 65, 85, and 105 in embplant_pt mode. Genome annotations were generated with GeSeq [38], with protein-coding sequences predicted using Chloë (v0.1.0) [39], tRNAs identified with tRNAscan-SE (v2.0.7) [40], and rRNAs annotated using HMMER [41]. All annotations were manually curated for accuracy. Circular genome maps illustrating structure and gene organization were created with OGDRAW (v1.3.1) [42].

### Comparative analyses and identification of inversion event

Structural rearrangements, particularly inversion events, were identified through a combination of visual inspection of the circular cp genome maps and comparative genomic analyses. Initial assessments involved *in silico* comparisons of gene order and orientation. To further validate inversion boundaries and identify associated repeat elements, BLAST-based sequence alignments [43] were conducted, and dot plot analyses were performed using BLAST [43] and re-DOT-able (v1.2) (https://github.com/s-andrews/redotable). The confirmed inversion events were visualized using Easyfig (version 2.2.5) [44], enabling a clear depiction of gene arrangement and breakpoint regions.

### Phylogenetic analysis

Phylogenetic reconstruction was conducted using whole cp genome sequences. For the subfamily Aurantioideae, 28 species were analyzed, while a broader Rutaceae phylogeny included 41 cp genomes, comprising the same 28 Aurantioideae species and 13 outgroup taxa representing other major Rutaceae lineages. Multiple sequence alignments were generated with HomBlocks (v1.0) [45] using default parameters, and poorly aligned regions were removed with trimAl (v1.4, revision 15) [46] under the -automated1 option. The best-fitting nucleotide substitution model for each dataset was selected using ModelTest-NG (v0.1.7) [47]. Maximum likelihood phylogenies were inferred with RAxML (v8.2.12) [48] and evaluated with 1000 bootstrap replicates. Resulting trees were visualized and explored using Dendroscope (v3.8.8) [49] to interpret evolutionary relationships and the placement of the sIR pair within a broader phylogenetic context.

### Detection of alternative cp genome structural forms

To investigate the presence of alternative structural forms of cp genomes, short-read mapping was performed. Raw sequencing reads were aligned to their respective assembled cp genomes using BWA-MEM (v0.7.18-r1243-dirty) with default parameters, with the -M option enabled to mark shorter split alignments as secondary, using two CPU threads [50]. Alignments were sorted and indexed with SAMtools v1.14 [51], and read depth was subsequently quantified using the SAMtools depth function. Mapped paired-end reads were visualized with the Integrative Genomics Viewer (IGV) (v2.19.1) [52] to facilitate the detection of structural variants.

Paired-end reads spanning the regions where HR occurred were quantified to evaluate the potential coexistence of alternative cp genome configurations. Candidate reads were defined by an expected insert size of approximately 22 kb and discordant orientation when aligned to the normal-type reference genome. Discordant paired-end reads were classified and analyzed using stringent criteria: at least one read of the pair had to overlap with either sIR-1 or sIR-2. In cases without direct overlap, one read was required to map within the 22 kb-HR region, while its mate mapped outside this region, in the vicinity of either sIR-1 or sIR-2. When these reads aligned to the inverted-type reference genome, the distance between mate pairs was required to be less than 500 bp.

In addition to fully aligned discordant paired-end reads, we examined partially aligned discordant reads mapped to the normal type reference genome, referred to as soft-clipped reads. These reads were extracted and remapped to regions flanking the opposite sIR arm to identify support for inverted cp genome configurations.

The total frequency of alternative structural forms in each species was calculated as the number of supporting reads divided by the mean depth at the 22 kb-HR region.

Secondary structures within each individual sIR were predicted using the Mfold web server https://www.unafold.org/mfold/applications/dna-folding-form.php [53].

### PCR validation of structural forms

To experimentally validate the presence of alternative cp genome structures, PCR assays were conducted using four specifically designed primers: F1 (5′-ACTCGTACACCGGATTAGCAATCCGRCGCTTTAAKCCACTCAG-3′ ), R1 (5′-GTCGAACAAGAGAMTCRGATGTAATCAAGA-3′), F2 (5′-ACTGTCAAGTCGATTCATCRTCGAGAATTC-3′), and R2 (5′-GTAARSCGGTCGATTATAGAAAAG-3′). These primers were designed to flank the repeat regions implicated in inversion events, enabling amplification of isoform-specific products. PCR reactions were set up in 20 µL volumes containing: 2 µL of 2 ng/µL template DNA, 10 µL of 2 × KOD FX Neo PCR buffer (TOYOBO, Japan), 4 µL of 2 mM dNTPs, 0.4 µL (1.0 U/µL) of KOD FX Neo polymerase (TOYOBO, Japan), 1.2 µL each of 5 µM forward and reverse primers, and nuclease-free water. Thermal cycling conditions consisted of an initial denaturation at 94°C for 2 min, followed by cycles of denaturation at 98°C for 10 s, annealing at 60°C for 30 s, and extension at 68°C for 30 s per kilobase. To ensure detection sensitivity, PCRs were performed with cycle numbers ranging from 25 to 40, with specific species subjected to 25, 30, 35, or 40 cycles depending on amplification efficiency and template quality. Amplicons were resolved on 1.5% (w/v) agarose gels alongside a 100 bp DNA ladder (Promega, USA) for size estimation.

To investigate the unexpected PCR amplicon observed in *Atalantia ceylanica*, gel bands were excised and purified using the Isospin Agarose Gel Extraction protocol (NIPPON GENE, Japan). The recovered products were subjected to Sanger sequencing using the Spectrum Compact CE System (Promega, USA) with the BigDye Terminator v3.1 Cycle Sequencing Kit (Thermo Fisher Scientific, USA).

### Use of generative AI

ChatGPT (https://chat.openai.com/) and Google Gemini (https://gemini.google.com/) were used to rewrite the manuscript into more appropriate English and proofread the English text. Subsequently, the authors verified the correctness of the revisions.

## Results

### Short inverted repeat-mediated inversion occurs at distinct phylogenetic positions in the cp genome

**Figure 1.**
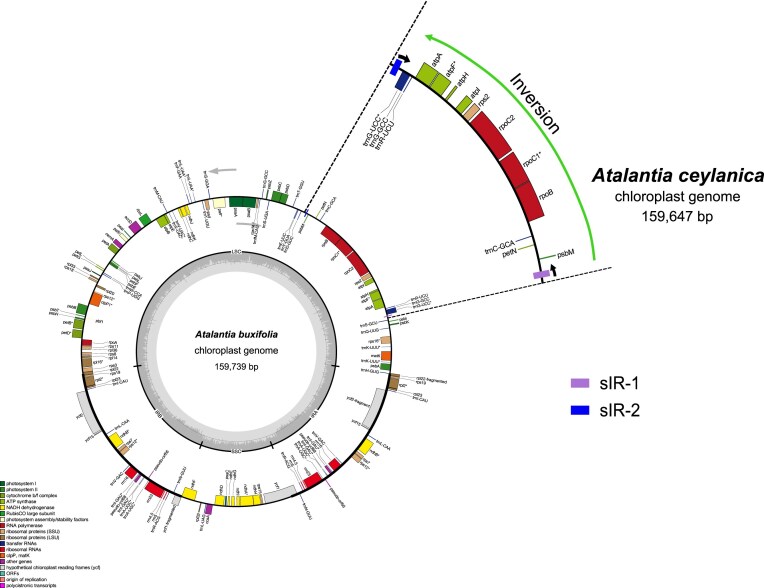
Structural comparison of the cp genome regions flanking *psbM* and *trnG-UCC*. The complete cp genome map of *Atalantia buxifolia* is shown as a reference, while only the homologous inverted region flanking *psbM* and *trnG-UCC* is depicted for *Atalantia ceylanica* to highlight the structural rearrangement. Light purple and blue boxes indicate the two arms of the sIR pair: sIR-1 located near *trnS-GCU* (light purple) and sIR-2 near *trnD-GUC* (blue). Genes containing introns are marked with an asterisk. IRA and IRB denote the inverted repeats; LSC, the large single-copy region; SSC, the small single-copy region. Gray arrows indicate transcriptional direction, and the innermost circle represents GC content.

A comprehensive comparative analysis of cp genome sequences was conducted across 28 species within the subfamily Aurantioideae, leveraging data from our previous study [21]. To investigate structural variation, circular cp genome maps were generated for each species based on manually curated annotations, and comparative analyses of the cp maps were subsequently performed. These comparisons consistently revealed a prominent large-scale inversion of approximately 22 kb within the LSC region, detected in *Atalantia ceylanica, Atalantia roxburghiana*, and *Naringi crenulata* (Fig. 1, Supplementary Fig. S1). Specifically, *Atalantia ceylanica* and *Atalantia roxburghiana* were compared with their close relative *Atalantia buxifolia*, while *Naringi crenulata* was compared with the closely related *Citropsis gabunensis* (Fig. 1, Supplementary Fig. S1). The boundaries of this inverted segment were flanked by regions proximal to the *psbM* and *trnG-UCC* genes (Fig. 1, Supplementary Fig. S1). Supporting this observation, dot plot analyses between each of the three species and their respective close relatives consistently exhibited a distinct inversion pattern spanning the region surrounding *psbM* and *trnG-UCC* (Supplementary Fig. S2).

**Figure 2.**
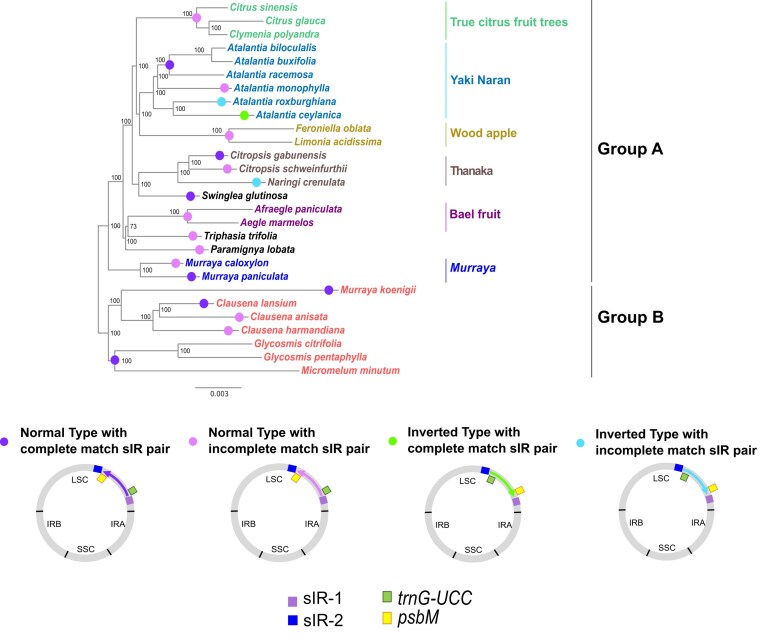
Distribution of normal and inverted cp genome types in an ML phylogenetic tree of 28 Aurantioideae species. The tree is based on complete cp genome sequences, with an alignment comprising 86 892 nucleotide positions, including 3707 parsimony-informative sites. Phylogenetic inference was conducted using RAxML under the GTRGAMMAIX model, selected as the optimal evolutionary model. The tree was rooted according to the position identified in the phylogeny of the Rutaceae family from our previous study [21]. Bootstrap support values (percentages from 1000 replicates) are shown at the nodes, and the scale bar represents genetic divergence in substitutions per site. Circular symbols highlight cp genome structural variation: purple indicates the normal type with a complete match sIR pair, pink indicates the normal type with an incomplete match sIR pair, light green represents the inverted type with a complete match sIR pair, and light blue represents the inverted type with an incomplete match sIR pair. Subgroups within Aurantioideae are indicated by different colors. The alignment (FASTA format) and tree (Newick format) files are provided in Supplementary Dataset 1.

**Figure 3.**
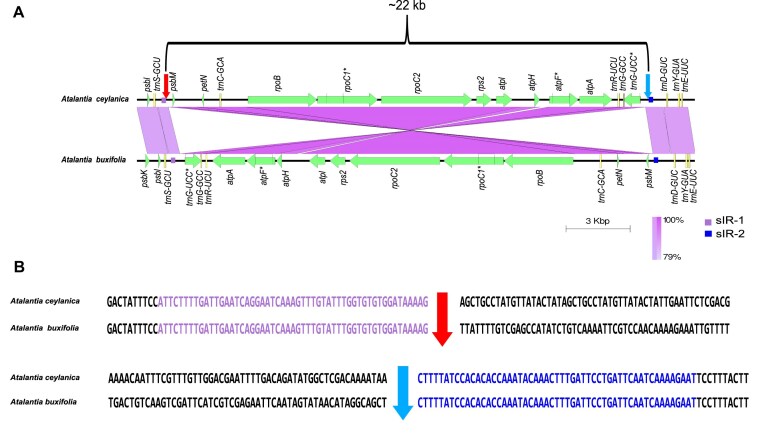
Comparative analysis of a flanking inversion in the *psbM–trnG-UCC* region of cp genomes. **(A)** Inversion visualization generated with Easyfig. Red and light blue arrows mark the inferred breakpoints. The gene arrangement of *Atalantia buxifolia* is shown as a reference and compared against the inversion observed in *Atalantia ceylanica*. Light purple and blue boxes indicate the two arms of the sIR pair, with sIR-1 located near *trnS-GCU* (light purple) and sIR-2 near *trnD-GUC* (blue). **(B)** The precise breakpoint of the inversion was identified within a 116 bp nucleotide segment. The 53 bp sequences of sIR-1 and sIR-2 at both ends of the inverted region are highlighted in light purple and blue, respectively.

To assess the evolutionary context of this inversion, we constructed an ML phylogenetic tree. Interestingly, the inversion appears to have originated through two independent events at distinct positions in the phylogeny, as indicated by light green and light blue circular symbols in Fig. 2, while the normal type was observed at many other positions, denoted by purple and pink circular symbols in the same figure (Fig. 2). Moreover, the inversion was located at identical nucleotide positions in all three species (Fig. 3, Supplementary Figs S3 and S4). Detailed sequence analysis of the inversion breakpoints revealed that one end contains a 53 bp sequence and the opposite end carries its corresponding inverted repeat, together forming an sIR pair flanking the inverted region (Figs 1 and 3, Supplementary Figs S1, S3, and S4). We designate these two arms of the repeat as sIR-1 and sIR-2, located proximally to the *trnS-GCU* and *trnD-GUC* genes, respectively (Fig. 1).

The sIR pair was consistently identified in Aurantioideae species, although in several taxa one arm displayed slight sequence variation caused by substitutions, insertions, or deletions (Fig. 2, Supplementary Table S2). As shown in Fig. 2, purple circular symbols indicate the normal form with a fully conserved sIR pair, while pink circular symbols correspond to the normal form with an incomplete match sIR pair. The inverted form was represented by light green circular symbols when the sIR pair was complete, and by light blue circular symbols when sequence mismatches slightly disrupted one arm. Sequence variation between the two sIRs was asymmetric: in some species (e.g. *Aegle marmelos*), sIR-1 contained substitutions while sIR-2 remained conserved, whereas in others (e.g. *Feroniella oblata*), sIR-2 showed variation and sIR-1 remained unchanged (Supplementary Table S2).

To further elucidate the distribution of this sIR pair, we extended our analysis to 13 species within the Rutaceae family, specifically those outside the Aurantioideae subfamily. Using the surrounding sequences of sIR-1 and sIR-2 from *Atalantia buxifolia* as references, we performed alignment comparisons for each species (Supplementary Figs S5 and  S6). In *Cneoridium dumosum*, for instance, the sIR-1 region contained an insertion, and its surrounding sequence exhibited low conservation compared with *Atalantia buxifolia* (Supplementary Fig. S5A). In contrast, the sIR-2 sequence showed a perfect match to that of *Atalantia buxifolia*, and its flanking region was highly conserved (Supplementary Fig. S5B). Across all non-Aurantioideae Rutaceae species, no well-conserved sIR pair was detected (Supplementary Figs S6 and S7). In most species, sIR-2 was clearly identifiable, either as a complete match or with only minor mismatches, and its surrounding region remained strongly conserved (Supplementary Figs S6 and S7). In contrast, sIR-1 showed substantial variability, frequently containing insertions and exhibiting reduced conservation in the flanking regions (Supplementary Figs S6 and S7). Moreover, in *Dictamnus albus, Psilopeganum sinense, Ruta graveolens, Thamnosma texana*, and *Zanthoxylum piperitum*, the entire sIR-1 region and its surrounding sequences were absent (Supplementary Figs S6 and S7).

Given the deeper conservation of sIR-2 relative to sIR-1 across outgroup taxa, we considered the possibility that sIR-2 may contain a characteristic sequence feature. To investigate this, we examined its potential to form secondary structures. In the majority of Aurantioideae species, sIR-2 is predicted to form a conserved 22 bp hairpin (TTTGATTCCTGATTCAATCAAA), consisting of a 7 bp stem and an 8-nucleotide loop (Supplementary Fig. S8, Supplementary Table S3). However, a few sequence divergences in *Paramignya lobata, Triphasia trifolia, Feroniella oblata*, and *Limonia acidissima* modified the base-pairing configuration (Supplementary Table S3). The GC content of sIR-2 in Aurantioideae (excluding these four divergent species) ranged from 28.3% to 32.08%, lower than the average GC level of their cp genomes (∼38%). Consistent with this low GC content, the stem of the predicted hairpin is composed primarily of A–T base pairs, indicating that the structure is likely unstable (Supplementary Fig. S8, Supplementary Tables S2 and S4). Notably, this 22 bp hairpin was not detected in any of the non-Aurantioideae species except *Cneoridium dumosum* and *Boenninghausenia albiflora* (Supplementary Table S3). A plastome-wide search further confirmed that this 22 bp motif does not occur elsewhere within Rutaceae cp genomes, indicating that it represents a structurally unique feature confined to the sIR pair.

### Short reads mapping reveals the presence of potential structural isoforms in Aurantioideae cp genomes

Across phylogenetically distant Aurantioideae species, we detected an identical inversion at the same locus of the cp genome, mediated either by a complete match sIR pair (light green in Fig. 2) or by an incomplete match sIR pair (light blue circles in Fig. 2), indicating two independent events. In contrast, many closely related species without any detectable inversion nevertheless harbored either a perfectly conserved sIR pair (purple circles in Fig. 2) or a pair with slight sequence divergence (pink circles in Fig. 2). These observations led us to propose that species harboring sIR pairs may contain two distinct cp genome configurations: one in the normal orientation and the other carrying the ∼22 kb inversion, with one configuration generally predominating and giving rise to the pattern we detected (Fig. 2). To test this hypothesis, we employed two complementary approaches: (1) bioinformatic analysis of short-read sequencing data and (2) experimental validation using a polymerase chain reaction (PCR)-based assay.

**Figure 4.**
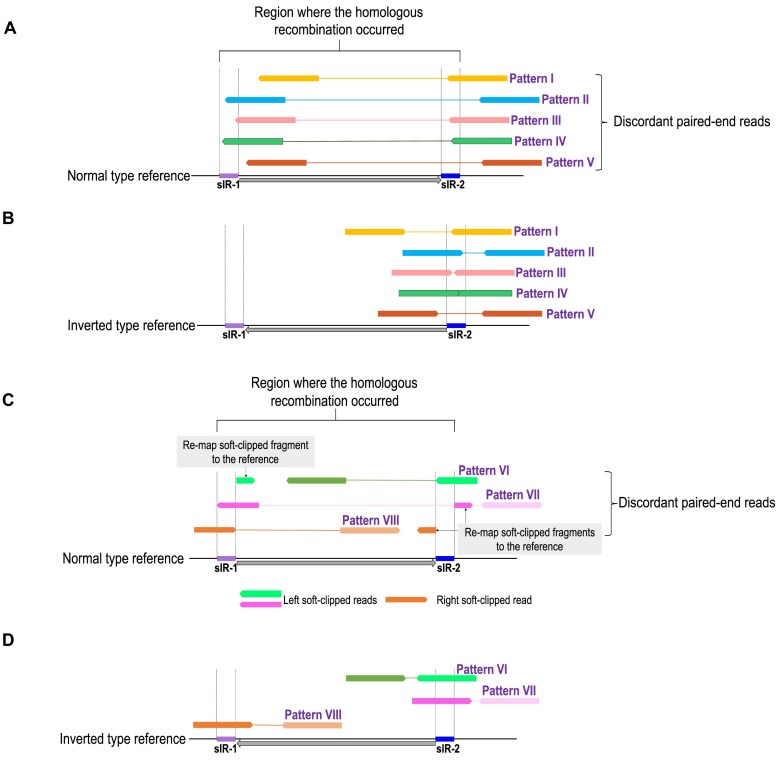
Short-read mapping patterns revealing alternative structural forms of the cp genome. Panels **(A)** and **(C)** show discordant paired-end reads aligned to the normal-type reference, focusing on the region where HR occurred, with panel **(C)** displaying soft-clipped reads. Panels **(B)** and **(D)** show the same reads mapped to the inverted-type reference, restoring proper orientation and complete alignment.

We reanalyzed short-read sequencing data from our previous study for all 28 species. Mapping depth was consistently high across both the whole plastome (173×–1 192×) and the 22 kb-HR region (171×–1 241×) (Supplementary Table S4), providing a robust foundation for downstream analyses. Mapped reads were visualized in the Integrative Genome Viewer (IGV), focusing on discordant paired-end reads with an expected insert size of approximately 22 kb spanning the region where HR occurred (Fig. 4). Candidate reads were classified and analyzed under stringent criteria: (1) at least one read of the pair overlapped with either sIR-1 or sIR-2, corresponding to Patterns I–VIII, excluding Pattern V; or (2) if no direct overlap occurred, one read mapped within the 22 kb-HR region while its mate mapped outside the region, near either sIR-1 or sIR-2 in the flanking sequence, corresponding to Pattern V. For reads aligned to the inverted-type reference genome, the span between mates was required to be less than 500 bp (Fig. 4). Analysis of these reads revealed eight distinct patterns supporting the presence of an inverted-type cp genome (Fig. 4).

**Figure 5.**
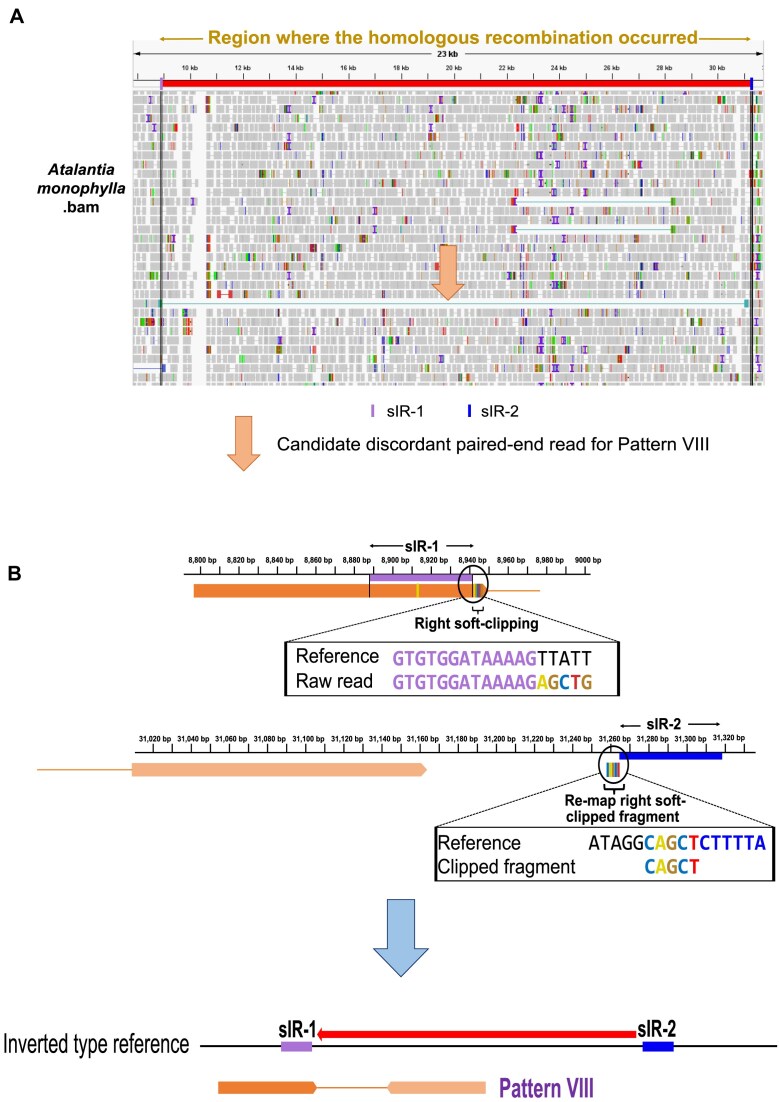
Selection and analysis of candidate paired-end reads exhibiting soft-clipping based on short-read mapping. **(A)** IGV visualization of short-read mapping in *Atalantia monophylla*. The upper panel shows genomic coordinates from the *de novo* assembled reference genome, while the lower panel displays the alignment of paired-end reads. The arrow indicates discordant candidate paired-end reads corresponding to Pattern VIII that were soft-clipped. The 53 bp sIR-1 and sIR-2 sequences located at the two ends of the inverted region are shown in light purple and blue, respectively. Mean read depth at the breakpoint positions was 523 × for sIR-1 and 578 × for sIR-2. **(B)** Illustration of soft-clipping at the sIR-1 region and remapping of the clipped fragments near sIR-2 in the normal-type reference. When mapped to the inverted-type reference, these fragments align fully in the correct orientation.

Five patterns (I–V) aligned either completely or with minor variation to the normal-type reference, but their paired-end orientations were discordant (Fig. 4A). When these same reads were mapped to the inverted-type reference, they aligned in the normal orientation, either perfectly or with minor variation, thereby supporting the presence of an inverted form (Fig. 4B). The remaining three patterns (VI–VIII) exhibited partial alignment at one end of the paired-end reads when mapped to the normal-type reference (Fig. 4C). The unaligned portion, referred to as a soft-clipped fragment, was remapped to the region surrounding the opposite sIR arm (Fig. 4C). In contrast, mapping to the inverted-type reference yielded full alignment of these reads (Fig. 4D). The presence of such soft-clipped reads thus provides direct evidence for the existence of the inverted form. *Atalantia monophylla*, which possesses a normal-type cp genome with an incompletely matching sIR pair, provides a clear example of this phenomenon corresponding to Pattern VIII (Fig. 5). In this case, the read was soft-clipped near the sIR-1 boundary in the normal reference, and the clipped fragment remapped near the sIR-2 boundary (Fig. 5B). On the inverted reference, the same read aligned seamlessly, demonstrating that the inversion resolves sequences that appear fragmented or discordant in the normal genome, thereby demonstrating the presence of the inverted form.

*Murraya koenigii, Atalantia buxifolia*, and *Atalantia racemosa*, each carrying a normal-type plastome with a perfectly matching sIR pair, exhibited exclusively fully aligned patterns. A single supporting read was detected for Pattern II in *Murraya koenigii*, Pattern III in *Atalantia buxifolia*, and Pattern V in *Atalantia racemosa*, corresponding to frequencies below 1% (Table 1, Supplementary Figs S9 and S10). Notably, *Atalantia monophylla* displayed a supporting read for the fully aligned Pattern V in addition to the previously described soft-clipped Pattern VIII, yielding a total frequency of 0.4% (Table 1, Supplementary Fig. S10). In contrast, *Afraegle paniculata*, which also carries a normal-type plastome but with an incomplete match sIR pair, exhibited six patterns (I, II, IV, V, VI, and VII). These included fully aligned reads, reads with slight variations such as nucleotide substitutions, short deletions or insertions, and soft-clipped reads with or without minor variation. In total, 35 reads supported these patterns, representing an estimated frequency of approximately 6.9% (Table 1, Supplementary Fig. S11). Collectively, these results provide preliminary evidence for the coexistence of two genome isoforms: the normal form and the alternative inverted form (Table 1).

**Table 1.** Number of discordant paired-end reads spanning the 22 kb-HR region and their estimated frequencies supporting the presence of alternative isoforms

Species	Number of discordant paired-end reads spanning the 22 kb-HR region for the observed pattern								Total number of discordant paired-end reads spanning the 22 kb-HR region	Total estimated frequency % based on mean depth for 22 kb-HR region
	I	II	III	IV	V	VI	VII	VIII		
Citrus glauca*	-	-	-	-	-	-	-	-	0	0
Atalantia monophylla*	-	-	-	-	1	-	-	1	2	0.38
*Atalantia ceylanica*	-	-	-	-	-	-	-	-	0	0
Atalantia roxburghiana*	-	-	-	-	-	-	-	-	0	0
*Atalantia buxifolia*	-	-	1	-	-	-	-	-	1	0.09
*Atalantia racemosa*	-	-	-	-	1	-	-	-	1	0.28
Feroniella oblata*	-	-	-	-	-	-	-	-	0	0
*Citropsis gabunensis*	-	-	-	-	-	-	-	-	0	0
Naringi crenulata*	-	-	-	-	-	-	-	-	0	0
*Swinglea glutinosa*	-	-	-	-	-	-	-	-	0	0
Afraegle paniculata*	9	2	-	2	11	9	2	-	35	6.9
Paramignya lobata*	-	-	-	-	-	-	-	-	0	0
Murraya caloxylon*	-	-	-	-	-	-	-	-	0	0
*Murraya koenigii*	-	1	-	-	-	-	-	-	1	0.13
Clausena anisata*	-	-	-	-	-	-	-	-	0	0
*Glycosmis citrifolia*	-	-	-	-	-	-	-	-	0	0
*Micromelum minutum*	-	-	-	-	-	-	-	-	0	0

*Species that have an incomplete match sIR pair

By contrast, no such patterns were detected in *Atalantia ceylanica, Atalantia roxburghiana*, or *Naringi crenulata*, where the inverted configuration represented the dominant form (Table 1). Similarly, in many other species, reads supporting the alternative configuration were not observed, regardless of whether the sIR pairs were completely identical or exhibited slight mismatches (Table 1, Supplementary Table S5).

### PCR-based assay confirms the coexistence of two distinct cp genome forms

**Figure 6.**
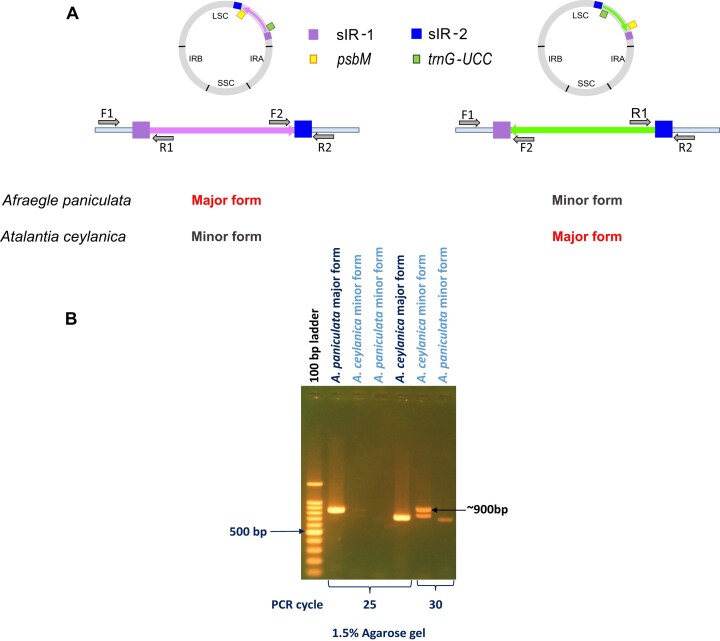
Coexistence of major and minor cp genome structural forms within individuals revealed by PCR-based assay. **(A)** Detection of both the normal and inverted cp genome configurations in *Afraegle paniculata* and *Atalantia ceylanica*, with the two forms appearing as major and minor types. Primer pair F1/R1 amplifies sIR-1, and F2/R2 amplifies sIR-2 in the major form of *Afraegle paniculata* and the minor form of *Atalantia ceylanica*. Conversely, F1/F2 amplifies sIR-1, and R1/R2 amplifies sIR-2 in the minor form of *Afraegle paniculata* and the major form of *Atalantia ceylanica*. **(B)** PCR-based verification of the coexistence of both structural isoforms using sIR-1 in *Afraegle paniculata* and *Atalantia ceylanica*. In *Atalantia ceylanica*, two bands appeared for the minor form, but the ∼900 bp band was validated as the correct isoform by Sanger sequencing. Primer combinations used for amplification are indicated for each sample and isoform: *A. paniculata* major form (F1/R1), *A. ceylanica* minor form (F1/R1), *A. paniculata* minor form (F1/F2), and *A. ceylanica* major form (F1/F2).

Mapping-based analysis revealed four distinct scenarios regarding alternative plastome configurations: (1) normal-type plastomes with an incompletely matched sIR pair showed a higher number of supporting reads (35 reads for *Afraegle paniculata* and 2 reads for *Atalantia monophylla*) for the inverted form; (2) normal-type plastomes with a fully matched sIR pair showed only a single supporting read for the inverted form; (3) normal-type plastomes with either complete or incomplete matched sIR pairs had no supporting reads for the inverted form; and (4) inverted-type plastomes, regardless of sIR match status, had no supporting reads for the normal form. These results raised two critical questions: whether the alternative configurations genuinely exist, and whether zero or single supporting reads indicate low-frequency structural variants below the detection threshold. To resolve this, we performed a PCR-based assay targeting the flanking regions of the repeated sequences associated with the putative inversion events (Fig. 6A). For this, four primers were designed to encompass each repeat region, and two primer combinations per repeat were employed to detect the alternative structural configurations (Fig. 6A).

With respect to the first scenario, mapping analysis of *Afraegle paniculata* revealed six distinct patterns supported by a total of 35 reads, despite the presence of incompletely matching sIRs, indicating the possible occurrence of an inverted cp genome isoform (Table 1, Supplementary Fig. S11). In this species, the typical non-inverted form was predominant, whereas the inverted form was present at a lower frequency (Fig. 6A). PCR amplification of sIR-1 at 25 cycles produced a strong ∼900 bp band corresponding to the major form. Increasing the cycle number to 30 allowed detection of an additional ∼700 bp band corresponding to the minor form, thereby confirming the coexistence of both structural types (Fig. 6B, Supplementary Table S6). Amplification of sIR-2 further supported the presence of the minor isoform, with a ∼350 bp band detectable at 25 cycles (Supplementary Fig. S12, Supplementary Table S6). Collectively, these results provide strong experimental validation of the mapping-based inference, confirming the coexistence of both major and minor cp genome isoforms in *Afraegle paniculata*.

In the second scenario, represented by *Murraya koenigii*, mapping-based analysis detected only a single supporting read, suggesting the potential presence of a minor inverted plastome configuration. This species possesses a perfectly conserved sIR pair. PCR assays targeting sIR-1 revealed a clear ∼900 bp fragment corresponding to the predominant major form at 30 amplification cycles. When extended to 35 cycles, an additional ∼700 bp fragment was detected, indicating the presence of the minor form (Supplementary Fig. S13, Supplementary Table S6). Further amplification using sIR-2 yielded a ∼400 bp fragment, providing independent confirmation of the minor inverted configuration (Supplementary Fig. S13, Supplementary Table S6).

The third scenario was exemplified by *Feroniella oblata*, which possesses a normal-type cp genome form as the predominant configuration with a non-identical sIR pair. Here, mapping analysis failed to detect any reads supporting the minor form (Supplementary Fig. S13, Supplementary Table S6). Nevertheless, PCR assays successfully amplified both major and minor structural forms, with band sizes consistent with the expected sizes (Supplementary Fig. S13,Supplementary Table S6), demonstrating that the minor form is present despite its absence in mapping data.

With respect to the fourth scenario, *Atalantia ceylanica* harbors a predominantly inverted cp genome form with a completely matched sIR pair. Mapping analysis did not detect reads supporting the normal-type arrangement (Fig. 6A, Table 1); however, PCR assays revealed that both genome configurations coexist. Amplification of sIR-1 at 25 cycles produced a strong ∼700 bp band corresponding to the major inverted form. At 30 cycles, two bands appeared at ∼900 and ∼800 bp, with the ∼900 bp band matching the expected size of the minor normal isoform (Fig. 6B, Supplementary Table S6). Sanger sequencing confirmed the identity of this band. Similarly, sIR-2 amplification detected the minor form, with a clear ∼350 bp band representing the major form visible at 20 cycles and a ∼200 bp band corresponding to the minor form appearing only after 35 cycles (Supplementary Fig. S12, Supplementary Table S6). These results confirm that *Atalantia ceylanica* harbors both normal and inverted cp genome isoform types.

In addition, *Atalantia roxburghiana* and *Naringi crenulata* possess an inverted-type cp genome form as the dominant configuration, but with incompletely matched sIR pairs (Fig. 2, Supplementary Table S2). Mapping analysis did not recover discordant paired-end reads (Table 1). In contrast, PCR assays detected bands corresponding to both major and minor structural arrangements, with fragment sizes matching the expected fragment lengths (Supplementary Fig. S14, Supplementary Table S6).

Together, these results demonstrate that both normal-type and inverted-type cp genome isoforms can coexist within a single species. The low number of supporting reads or absence of mapping support for the minor form likely reflects its very low abundance, which falls below the detection threshold rather than the true absence of the alternative configuration.

## Discussion

In this study, we uncovered evidence of a dynamic and ongoing structural rearrangement in the cp genomes of the subfamily Aurantioideae, providing a compelling snapshot of genome evolution in action. Our results delineate a multistep process: (1) the emergence of a pair of sIRs initiates structural instability; (2) these repeats act as substrates for intramolecular HR, resulting in a ∼22 kb inversion within the LSC region; (3) this recombination event generates structural heteroplasmy, with distinct genomic configurations coexisting within a single individual; and (4) the minor isoform has progressed toward dominance, likely influenced by genetic drift. The presence of these intermediate states of genomic flux, where alternative structural forms coexist and shift in frequency, highlights a period of heightened instability and demonstrates that such structural changes are not sudden but occur gradually over time. Unlike previous studies that primarily document fixed cp genome rearrangements [29, 30, 54], our findings highlight a unique instance of ongoing sIR-mediated structural heteroplasmy, revealing a previously underrecognized mechanism contributing to plastome evolution and variability.

Focusing on step 1, our investigation consistently identified an sIR pair, found in either a complete or an incomplete match, precisely at the breakpoints of a recurrent genomic inversion across all examined Aurantioideae species. Comparative analysis with outgroup taxa from the broader Rutaceae family revealed a notable evolutionary asymmetry: sIR-2 is highly conserved, whereas sIR-1 is either entirely absent or highly divergent, suggesting that the sIR pair first emerged within a common ancestor of the Aurantioideae. Furthermore, the presence of a conserved 22 bp hairpin within sIR-2, shared among the common ancestor of Aurantioideae, *Cneoridium dumosum*, and *Boenninghausenia albiflora*, provides a likely mechanistic basis for the subsequent emergence of sIR-1. Although energetically unstable, this hairpin structure may occasionally form during DNA replication, causing polymerase stalling events [55, 56]. Such stalling could promote backward realignment of the nascent strand, resulting in recopying of the entire 53 bp sIR-2 sequence. This slippage-mediated duplication would generate a second sIR copy, which could then be inserted at a distal genomic position through replication-fork instability or template switching. In Aurantioideae, this duplicated copy became positioned ∼22 kb from the ancestral site, forming sIR-1. The resulting sIR pair then provided the substrate for intramolecular recombination, enabling the recurrent ∼22 kb inversion observed across the group. This repeat pair may confer an advantage by facilitating genomic plasticity and promoting structural flexibility, thereby contributing to the diversification and evolutionary success of the Aurantioideae. Alternatively, the repeat pair might represent a nearly neutral feature with no direct adaptive value. In this case, its evolutionary fate would not be shaped by selection but rather by stochastic processes, where its retention or loss within a lineage is largely determined by the random effects of genetic drift.

In relation to step 2, our study provides compelling evidence that the sIR pair functions as a substrate for intramolecular HR, driving the observed ∼22 kb inversion. Intramolecular HR, a recombination between homologous DNA sequences [57], frequently underlies structural rearrangements in plastid genomes and is particularly prevalent in cp genomes due to the presence of large IRs typically ranging from 10 to 30 kb in length [25]. Recombination between these IRs results in the inversion of the LSC and SSC regions, a phenomenon commonly referred to as “flip-flop” recombination [25, 26]. This rapid and frequent process explains the widespread observation of cp genome isomers differing in SSC orientation. Beyond the canonical IRs, sIRs can also mediate inversions due to their relative abundance in plastomes [30, 58]. Indeed, a previous study reported widespread small inversions of 5–50 bp in both monocots and dicots, facilitated by 11–24 bp inverted repeats at the inversion boundaries, which form stem-loop hairpin structures that undergo HR and generate flip-flop orientations [30]. Larger inversions further illustrate the mechanistic potential of sIRs; for instance, a 34 kb inversion in *Calocedrus macrolepis* is mediated by 11 bp inverted repeats at the fragment boundaries [59], while a 36 kb inversion in legumes is associated with 29 bp sIRs [60]. Collectively, these examples demonstrate that sIRs, even when not associated with canonical large IRs, can serve as effective substrates for intramolecular HR and drive significant structural rearrangements. The mechanism we propose is conceptually analogous to non-allelic homologous recombination (NAHR) described in human genomic disorders, where homologous but non-allelic repeats misalign and recombine [61]. In our study, the 53 bp sIR pair functions as non-allelic inverted repeats of sufficient length to support RecA-like intramolecular HR, thereby generating the recurrent ∼22 kb inversion. Moreover, the hairpin structures formed independently by both sIR-1 and sIR-2 likely enhance recombination propensity by inducing local replication-fork stalling and transient single-stranded DNA exposure, predisposing the region to recombination. Together, these observations indicate that the sIR pair in Aurantioideae possesses sufficient length to mediate the observed inversion, underscoring the broader role of sIR-driven HR in plastome structural evolution.

The confirmed coexistence of distinct plastome isoforms, as outlined in step 3, provides direct evidence of structural heteroplasmy generated by a large-scale inversion mediated by an sIR pair. Heteroplasmy in organellar genomes can be broadly classified into point heteroplasmy, reflecting nucleotide-level variation, and length or structural heteroplasmy, arising from large-scale genomic rearrangements [62]. Within the subfamily Aurantioideae, plastome heteroplasmy has previously been reported in *Citrus*, but it was attributed to biparental inheritance, resulting in point heteroplasmy rather than structural heteroplasmy [63]. In plants more broadly, structural heteroplasmy is most frequently explained by flip-flop recombination between the two large IRs, which inverts the SSC region and generates two alternative haplotypes that are typically maintained at near-equal frequencies [22, 25, 26]. By contrast, structural heteroplasmy mediated by sIRs has been considered rare. For example, in the genus *Juniperus*, a 36 kb inversion flanked by ∼250 bp inverted repeats generates alternative plastome haplotypes, but these are detected only at very low frequencies, such as 5.0% in *J. scopulorum* and 0.8% in *J. monosperma* [32]. Many previous studies have reported that sIRs mediate inversion events that generate multiple cp genome forms within a genus; however, among these alternative forms, one configuration usually becomes stabilized and fixed, resulting in the presence of distinct plastome forms across an intraspecific population rather than their coexistence within a single individual [31, 54, 59, 60]. In sharp contrast, our findings provide the first clear evidence that sIR-associated structural heteroplasmy is not an isolated or transient phenomenon but extends across multiple members of a single subfamily, thereby uncovering an unprecedented dimension of plastome structural diversity and offering novel insights into the evolutionary dynamics of structural rearrangements at a broader taxonomic scale. Importantly, the Aurantioideae crown is estimated to have originated in the early Miocene (12.1–28.2 Ma) [64], indicating that this structural heteroplasmy is not a recent development but a deeply conserved feature. It has persisted since the emergence of the sIR pair in the common ancestor of Aurantioideae, reflecting the long-term maintenance of alternative plastome configurations over 12–28 million years.

As the fourth step in the process, our results demonstrate that the minor plastome isoform has become dominant in two independent phylogenetic lineages, suggesting that random genetic drift is the primary force driving these shifts. Random genetic drift is a well-established evolutionary mechanism shaping mitochondrial DNA (mtDNA) inheritance in animals, including humans, where heteroplasmy and drift cause stochastic fluctuations in allele frequencies and influence the manifestation of mtDNA-related diseases [65–67]. For example, studies of multigenerational human families have shown that mtDNA heteroplasmy undergoes severe germline bottlenecks, producing dramatic random shifts in allele frequencies between generations, while continued drift in oocytes during maternal aging further alters heteroplasmy levels [65]. By analogy, cp genome inheritance is also subject to bottlenecks, where only a limited number of genome copies are transmitted, thereby amplifying the effects of drift and enabling rare structural variants to rise in frequency or even reach fixation. In addition, the number of cp DNA copies within a cell varies with developmental and physiological state [68]. During active photosynthesis, cp DNA copy numbers are high, whereas in the absence of light, plastids differentiate into etioplasts or proplastids with markedly reduced cp DNA content [68, 69]. These reductions may act as additional bottlenecks, and in such contexts, the fate of alternative plastome variants is governed largely by random genetic drift. This process may allow rare structural forms to become enriched and, upon conversion to functional chloroplasts, potentially dominant. Together, these mechanisms provide a plausible explanation for why the inverted plastome form, typically present only as a minor variant in other Aurantioideae species, has become established as the predominant configuration in two phylogenetically distinct lineages over evolutionary history.

Our results revealed a markedly higher number of supporting reads for the inverted plastome configuration in *Afraegle paniculata* (35 reads; 6.9%) compared with all other Aurantioideae species examined. Two factors may explain this elevated frequency. First, as discussed in the earlier paragraph, random genetic drift may have increased the representation of the minor inverted configuration in *Afraegle paniculata*, leading to a higher detectable proportion than in other species. Second, the elevated presence of the minor isoform may reflect greater intrinsic structural instability in this lineage. Species-specific differences in replication dynamics [70], plastid DNA repair and recombination processes [71], and the influence of nuclear and cytoplasmic interactions, particularly because most proteins involved in plastome maintenance are nuclear encoded [72], may collectively promote more frequent generation or slower resolution of alternative plastome configurations.

The ongoing structural rearrangements of the cp genome observed in our study are not sudden events but occur gradually over evolutionary time. By contrast, structural changes in the nuclear genome, such as large chromosomal rearrangements or mutations, can arise rapidly through mechanisms including replication errors, double-strand breaks, or transposable element activity [73–75]. For example, chromosome breaks induced by radiation or chemical agents can result in segments of DNA detaching and incorrectly reattaching to other chromosomes, producing deletions, duplications, or translocations within a short timeframe [74]. Such abrupt events can immediately alter nuclear genome architecture and, if DNA repair is faulty, may contribute to diseases such as human cancer [75]. The processes underlying structural changes in the cp and nuclear genomes are therefore governed by fundamentally distinct molecular mechanisms, with plastid changes accumulating slowly and often being regulated, while nuclear changes can occur rapidly and stochastically.

Our study demonstrates that a rapid and cost-effective workflow using short-read sequencing data can successfully uncover significant, previously unreported structural dynamics in plastid genomes. This approach proved highly sensitive, enabling the detection of alternative isoforms even at extremely low frequencies, where, in some cases, only a single paired-end read spanned the inversion breakpoint. Crucially, follow-up PCR-based validation confirmed that these low read counts were not sequencing artifacts but represented genuine, rare isomeric forms of the plastome. These findings establish our workflow as a highly sensitive and practical tool for resolving complex cp genome architectures. Its scalability and reliance on widely accessible public short-read datasets make it a powerful method, facilitating broad-scale comparative and population-level investigations of plastome structural diversity. Importantly, our findings highlight the untapped potential of raw sequencing reads, which remain underutilized in standard assembly pipelines, underscoring the value of mining these datasets to uncover rare structural variants and fully capture plastome complexity. However, we acknowledge that long-read sequencing platforms such as PacBio HiFi and Oxford Nanopore Technologies (ONT) would offer substantial advantages, as their extended read lengths can span the entire ∼22 kb inversion and both sIR-defined breakpoints within a single molecule, enabling direct and unambiguous haplotype resolution [76]. Likewise, genomic optical mapping (e.g. Bionano) could independently validate large-scale structural configurations and visualize alternative plastome conformations at the single-molecule level [76]. Such capabilities are not achievable with short-read data, which cannot bridge repeat-mediated breakpoints or fully resolve complex plastome rearrangements.

In conclusion, our study demonstrates that the emergence of an sIR pair drives ongoing structural rearrangements in cp genomes across the subfamily Aurantioideae, resulting in structural heteroplasmy detectable from short-read sequencing data. These findings provide critical insights into the dynamic architecture of cp genomes and the molecular mechanisms underlying plastome rearrangements, while also highlighting the untapped potential of short-read data to resolve fine-scale structural variation. Looking forward, systematic mining of publicly available short-read datasets may uncover additional overlooked cases of cp genome rearrangements, offering new opportunities to illuminate the evolutionary dynamics and plasticity of plant organellar genomes.

## Supplementary Material

gkag117_Supplemental_Files

## Data Availability

Raw sequencing data and the assembled, annotated cp genome sequences were obtained from our previous study [21]. The raw sequencing data have been deposited in the DDBJ Sequence Read Archive under accession number DRA017638. The cp genome sequences assembled and annotated from these data are available in the DDBJ/EMBL/GenBank databases under accession numbers LC794878–LC794908. A complete list of datasets used in this analysis is provided in Supplementary Table S1. The alignments and tree data used in this study are included in Supplementary Datasets 1 and 2.
