# Supplementary material for: Ongoing structural changes highlight the dynamic nature of chloroplast genomes
Source: Nucleic Acids Res. 2026 Feb 24;54(4):gkag117. doi: 10.1093/nar/gkag117 (PMC12928735; doi:10.1093/nar/gkag117)
Supplement: gkag117_Supplemental_Files [file Supplementary_Figures.pdf › Supplementary_Figures.pdf]

Supplementary Figure Legends

Supplementary Figure S1. Structural comparison of cp genome regions flanking *psbM* and *trnG-UCC*

The complete cp genomes of *Atalantia buxifolia* (A) and *Citropsis gabunensis* (B) are shown as references, while only the homologous inverted regions flanking *psbM* and *trnG-UCC* are displayed for *Atalantia roxburghiana* and *Naringi crenulata*, respectively, to highlight the structural rearrangements. Light purple and blue boxes indicate the two arms of the sIR pair: sIR-1 located near *trnS-GCU* (light purple) and sIR-2 near *trnD-GUC* (blue). Genes containing introns are marked with an asterisk. IRA and IRB denote the inverted repeats; LSC, large single-copy region; SSC, small single-copy region. Gray arrows indicate the direction of transcription, and the innermost circle represents GC content.

Supplementary Figure S2. Comparative dot plot analyses of cp genomes

Dot plots were generated to visualize sequence similarity and detect potential structural rearrangements between *Atalantia ceylanica*, *Atalantia roxburghiana*, *Naringi crenulata*, and the reference genomes of *Atalantia buxifolia* and *Citropsis gabunensis*. (A) *A. ceylanica* vs. *A. buxifolia*; (B) *A. roxburghiana* vs. *A. buxifolia*; (C) *N. crenulata* vs. *C. gabunensis*.

Supplementary Figure S3. Inversion in the *psbM–trnG-UCC* region of *Atalantia roxburghiana*. (A) Visualization of the inversion using Easyfig; red and light blue arrows indicate the predicted breakpoints. The gene arrangement of *Atalantia buxifolia* is shown as a reference representing the normal-type cp genome. Light purple and blue boxes indicate the two arms of the sIR pair, with sIR-1 located near *trnS-GCU* (light purple) and sIR-2 near *trnD-GUC* (blue). (B) The inversion breakpoint was precisely localized within a 116-bp segment, flanked by 53-bp sequences of sIR-1 and sIR-2 at both ends of the inverted region, highlighted in light purple and blue, respectively.

Supplementary Figure S4. Inversion in the *psbM–trnG-UCC* region of *Naringi crenulata*. (A) Easyfig visualization of the inversion, with red and light blue arrows marking the predicted breakpoints. The gene order in *Citropsis gabunensis* is shown as a reference representing the normal-

type cp genome. Light purple and blue boxes indicate the two arms of the sIR pair, with sIR-1 located near *trnS-GCU* (light purple) and sIR-2 near *trnD-GUC* (blue). (B) The inversion breakpoint was precisely localized within a 116-bp segment, flanked by 53-bp sequences of sIR-1 and sIR-2 at both ends of the inverted region, highlighted in light purple and blue, respectively.

Supplementary Figure S5. Analysis of the sequences adjacent to the sIR pair in *Cneoridium dumosum, an* outgroup of Aurantioideae, referenced to *Atalantia buxifolia*; panel (A) depicts the region around sIR-1, and panel (B) depicts the region around sIR-2.

Supplementary Figure S6. Comparison of the genomic regions surrounding the sIR pair in outgroup members of Aurantioideae, using *Atalantia buxifolia* as a reference, with panel (A) showing sIR-1 and panel (B) showing sIR-2.

Supplementary Figure S7. Distribution of the sIR pair in a phylogenetic context, including 13 outgroup members of the Rutaceae family.

The phylogenetic tree was inferred from complete cp genome sequences (alignment length: 77,491 bp; parsimony-informative sites: 9,228) using RAxML under the GTRGAMMAIX model, identified as the best-fit evolutionary model. Bootstrap support values (percentages from 1,000 replicates) are shown at the nodes, and the scale bar represents substitutions per site. Circular symbols denote cp genome structural variation: purple indicates the normal type with a complete match sIR pair, pink indicates the normal type with an incomplete match sIR pair, light green represents the inverted type with a complete match sIR pair, light blue represents the inverted type with an incomplete match sIR pair, teal indicates the normal type with absence of the sIR-1 region and its surrounding sequences, and dark yellow indicates the normal type with a highly divergent sIR-1 and a less conserved surrounding region. Subgroups within Aurantioideae are indicated by different colors. The alignment (FASTA) and tree (Newick) files are provided in Supplementary Dataset 2.

Supplementary Figure S8. Predicted hairpin secondary structure within the sIR-2
ΔG – Estimated minimum free energy, Tm – Melting temperature

Supplementary Figure S9. Selection of candidate abnormal paired-end reads in *Murraya koenigii* and *Atalantia buxifolia* using IGV mapping.

(A) IGV visualization of *Murraya koenigii*. (B) IGV visualization of *Atalantia buxifolia.* Arrows indicate abnormal candidate paired-end reads corresponding to patterns. In each panel, the upper track shows genomic coordinates from the *de novo* assembled reference genome, and the lower track displays the alignment of paired-end reads. The 53-bp sIR-1 and sIR-2 sequences located at the two ends of the inverted region are shown in light purple and blue, respectively. Mean read depth at the breakpoint positions was 799× (sIR-1) and 731× (sIR-2) in *Murraya koenigii*, and 1178 × (sIR-1) and 939× (sIR-2) in *Atalantia buxifolia*.

Supplementary Figure S10. Selection of candidate abnormal paired-end reads in *Atalantia monophylla* and *Atalantia racemosa* using IGV mapping.

(A) IGV visualization of *Atalantia monophylla*. (B) IGV visualization of *Atalantia racemosa.* Arrows indicate abnormal candidate paired-end reads corresponding to patterns. The upper track represents genomic coordinates from the *de novo* assembled reference genome, while the lower track shows the alignment of paired-end reads. The 53-bp sIR-1 and sIR-2 sequences located at the two ends of the inverted region are shown in light purple and blue, respectively. Mean read depth at the breakpoint positions was 523× (sIR-1) and 578× (sIR-2) in *Atalantia monophylla*, and 283× (sIR-1) and 310× (sIR-2) in *Atalantia racemosa***.**

Supplementary Figure S11. Selection of candidate abnormal paired-end reads in *Afraegle paniculata* using IGV mapping.

Arrows indicate abnormal candidate paired-end reads corresponding to patterns. The upper track represents genomic coordinates from the *de novo* assembled reference genome, while the lower track shows the alignment of paired-end reads. The 53-bp sIR-1 and sIR-2 sequences marking the two ends of the inverted region are highlighted in light purple and blue, respectively. Mean read depth at the breakpoint positions is 579× for sIR-1 and 528× for sIR-2.

Supplementary Figure S12. PCR validation of two structural isoforms in *Afraegle paniculata* and *Atalantia ceylanica* (sIR-2 region)

PCR amplification was performed using two primer combinations to detect both isoforms associated with sIR-2. Gel images show amplification products generated using (A) the F2/R2 primer pair for the *A. paniculata* major form and *A. ceylanica* minor form, and (B) the R1/R2 primer pair for the *A. paniculata* minor form and *A. ceylanica* major form.

Supplementary Figure S13. PCR validation of two structural isoforms in *Murraya koengii* and *Feroniella oblata*

PCR was used to confirm the presence of alternative plastome configurations at the sIR-1 and sIR-2 regions. Gel images show amplification products targeting: (A) sIR-1, using the F1/R1 primer pair for the *M. koengii* major form and *F. oblata* major form, and the F1/F2 primer pair for the *M. koengii* minor form and *F. oblata* minor form; and (B) sIR-2, using the F2/R2 primer pair for the *M. koengii* major form and *F. oblata* major form, and the R1/R2 primer pair for the *M. koengii* minor form and *F. oblata* minor form.

Supplementary Figure S14. PCR validation of two structural isoforms in *Naringi crenulata* and *Atalantia roxburghiana*

PCR was conducted to detect isoforms associated with both sIR-1 and sIR-2 regions. Gel images show amplification products targeting: (A) sIR-1, using the F1/R1 primer pair for the *N. crenulata* and *A. roxburghiana* minor forms, and the F1/F2 primer pair for the *N. crenulata* and *A. roxburghiana* major forms; and (B) sIR-2, using the F2/R2 primer pair for the *N. crenulata* and *A. roxburghiana* minor forms, and the R1/R2 primer pair for the *N. crenulata* and *A. roxburghiana* major forms.

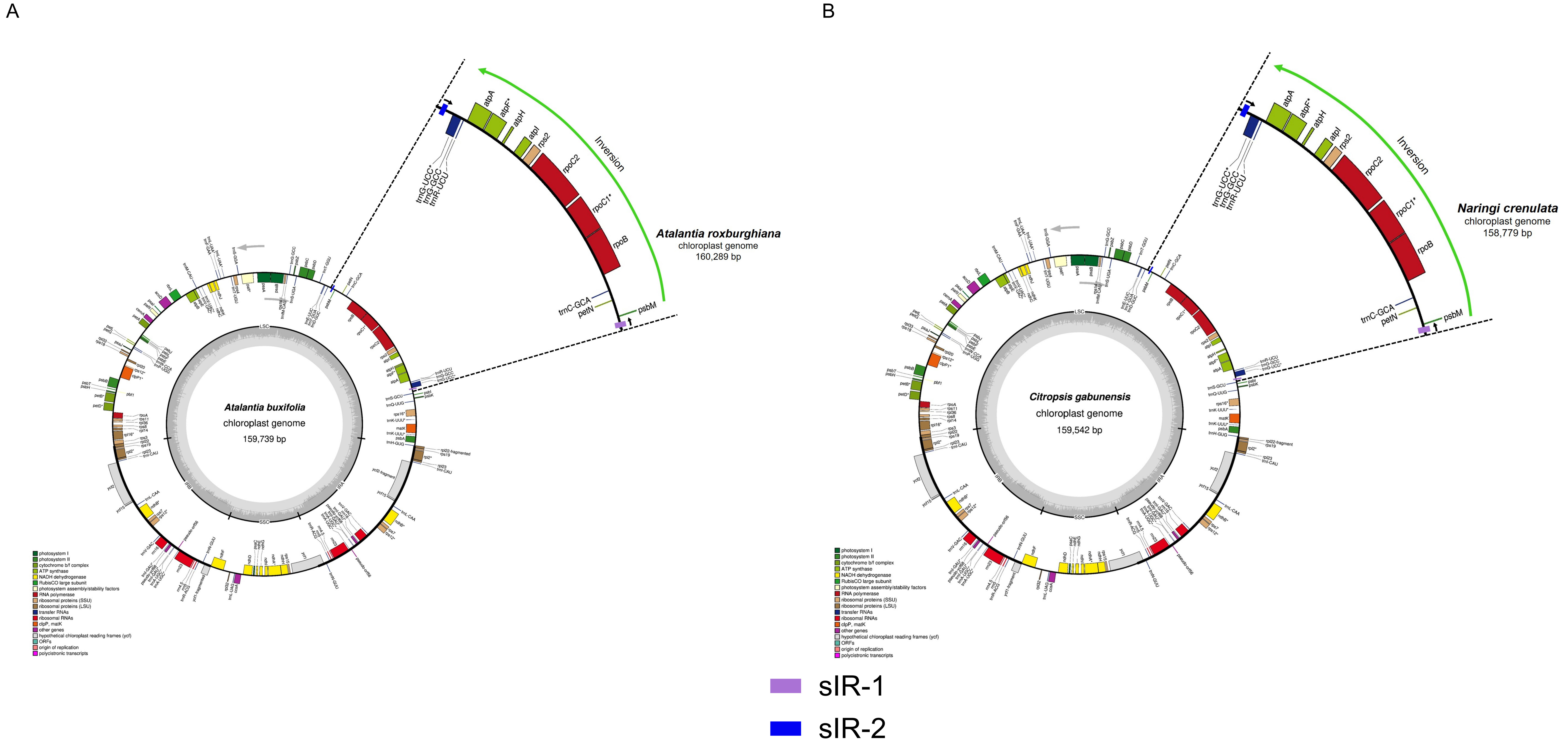
A
B
Inversion
Atalantia roxburghiana
chloroplast genome
160,289 bp
atpA
atpF*
atpH
atpI
rps2
rpoC2
rpoC1*
rpoB
trnG-UCC*
trnG-GCC
trnR-UCU
trnC-GCA
petN
psbM
Atalantia buxifolia
chloroplast genome
159,739 bp
Naringi crenulata
chloroplast genome
158,779 bp
Citropsis gabunensis
chloroplast genome
159,542 bp
LSC
SSC
IRA
IRB
sIR-1
sIR-2

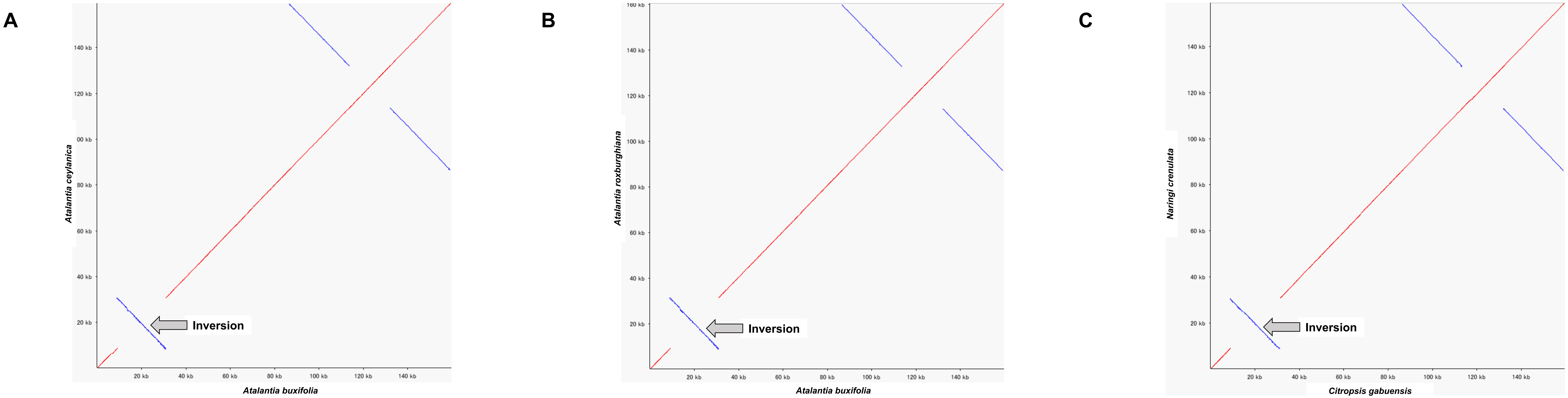
A
Inversion
Atalantia ceylanica
Atalantia buxifolia
B
Inversion
Atalantia roxburghiana
Atalantia buxifolia
C
Inversion
Naringi crenulata
Citropsis gabuensis
20 kb
40 kb
60 kb
80 kb
100 kb
120 kb
140 kb
160 kb

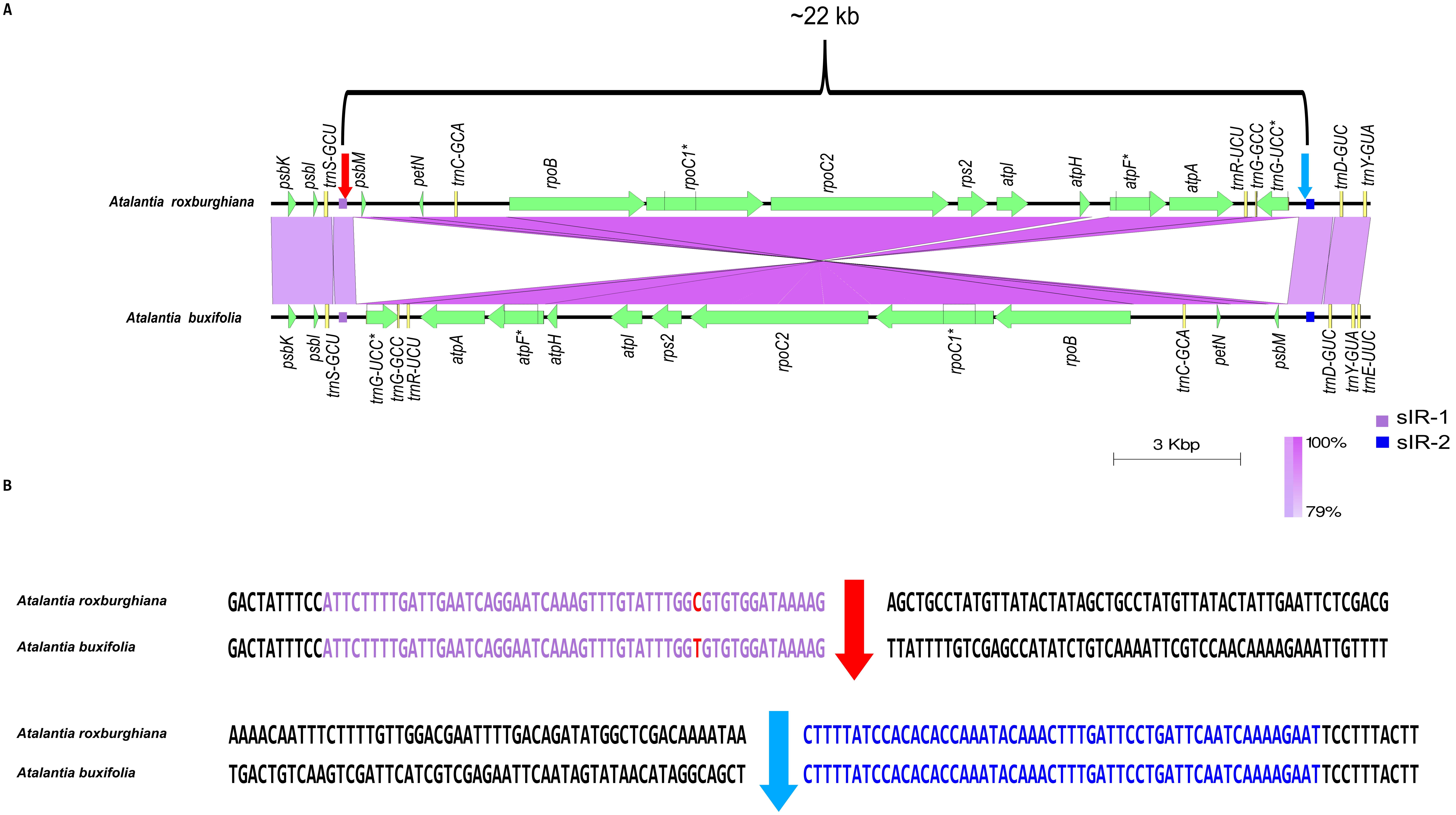
A
~22 kb
Atalantia roxburghiana
psbK
psbI
trnS-GCU
psbM
petN
trnC-GCA
rpoB
rpoC1*
rpoC2
rps2
atpI
atpH
atpF*
atpA
trnR-UCU
trnG-GCC
trnG-UCC*
trnD-GUC
trnY-GUA
Atalantia buxifolia
psbK
psbI
trnS-GCU
trnG-UCC*
trnG-GCC
trnR-UCU
atpA
atpF*
atpH
atpI
rps2
rpoC2
rpoC1*
rpoB
trnC-GCA
petN
psbM
trnD-GUC
trnY-GUA
trnE-UUC
3 Kbp
100%
79%
sIR-1
sIR-2
B
Atalantia roxburghiana
GACTATTTCCATTCTTTTGATTGAATCAGGAATCAAAGTTTGTATTTGGCGTGTGGATAAAAG
AGCTGCCTATGTTATACTATAGCTGCCTATGTTATACTATTGAATTCTCGACG
Atalantia buxifolia
GACTATTTCCATTCTTTTGATTGAATCAGGAATCAAAGTTTGTATTTGGTGTGTGGATAAAAG
TTATTTTGTCGAGCCATATCTGTCAAAATTCGTCCAACAAAAGAAATTGTTTT
Atalantia roxburghiana
AAAACAATTTCTTTTGTTGGACGAATTTTGACAGATATGGCTCGACAAAATAA
CTTTTATCCACACACCAAATACAAACTTTGATTCCTGATTCAATCAAAAGAATTCCTTTACTT
Atalantia buxifolia
TGACTGTCAAGTCGATTCATCGTCGAGAATTCAATAGTATAACATAGGCAGCT
CTTTTATCCACACACCAAATACAAACTTTGATTCCTGATTCAATCAAAAGAATTCCTTTACTT

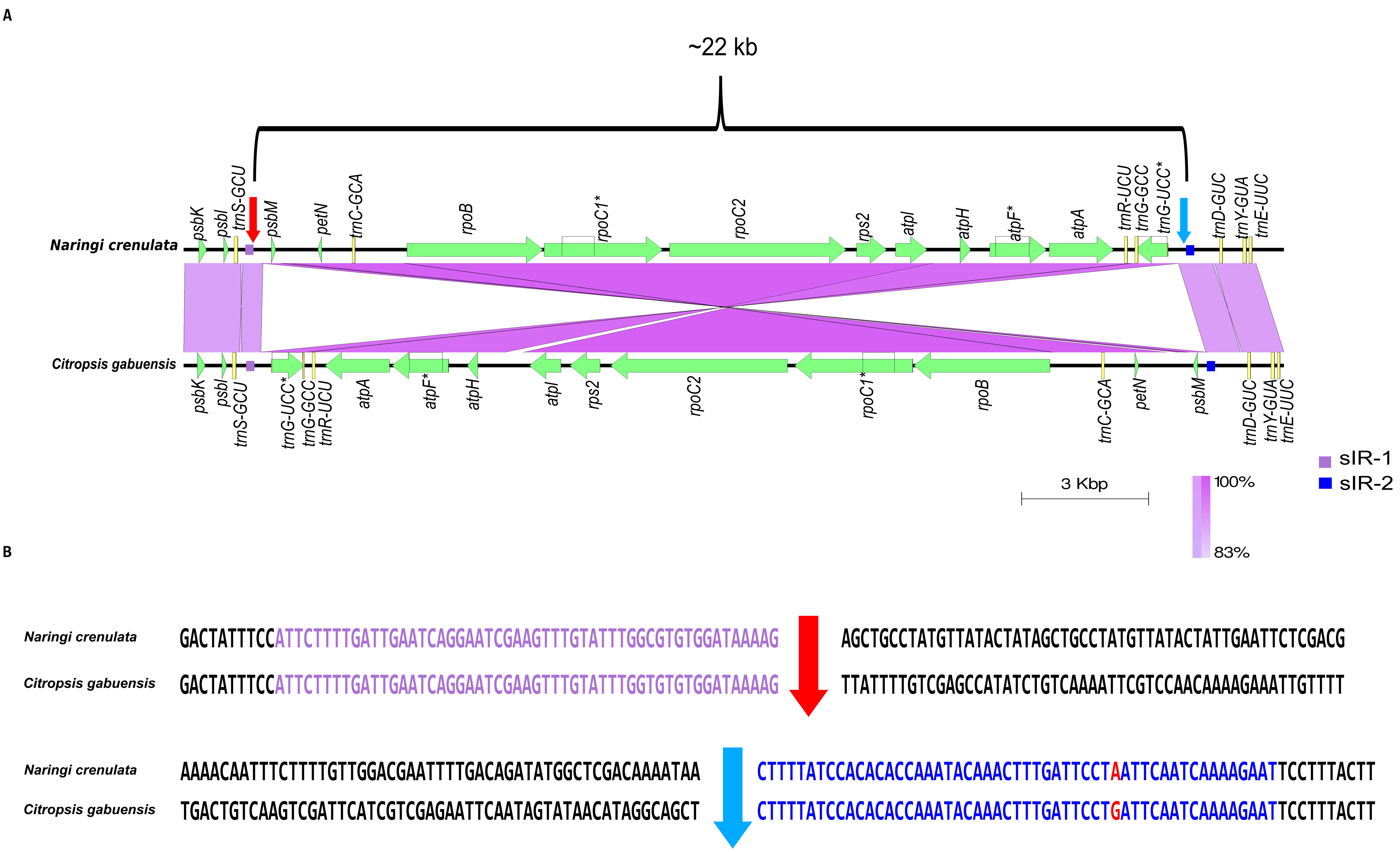

A
~22 kb
Naringi crenulata
Citropsis gabuensis
psbK
psbI
trnS-GCU
psbM
petN
trnC-GCA
rpoB
rpoC1*
rpoC2
rps2
atpI
atpH
atpF*
atpA
trnR-UCU
trnG-GCC
trnG-UCC*
trnD-GUC
trnY-GUA
trnE-UUC
sIR-1
sIR-2
3 Kbp
100%
83%
B
Naringi crenulata GACTATTTCCATTCTTTTGATTGAATCAGGAATCGAAGTTTGTATTTGGCGTGTGGATAAAAG AGCTGCCTATGTTATACTATAGCTGCCTATGTTATACTATTGAATTCTCGACG
Citropsis gabuensis GACTATTTCCATTCTTTTGATTGAATCAGGAATCGAAGTTTGTATTTGGTGTGTGGATAAAAG TTATTTTGTCGAGCCATATCTGTCAAAATTCGTCCAACAAAAGAAATTGTTTT
Naringi crenulata AAAACAATTTCTTTTGTTGGACGAATTTTGACAGATATGGCTCGACAAAATAA CTTTTATCCACACACCAAATACAAACTTTGATTCCTAATTCAATCAAAAGAATTCCTTTACTT
Citropsis gabuensis TGACTGTCAAGTCGATTCATCGTCGAGAATTCAATAGTATAACATAGGCAGCT CTTTTATCCACACACCAAATACAAACTTTGATTCCTGATTCAATCAAAAGAATTCCTTTACTT

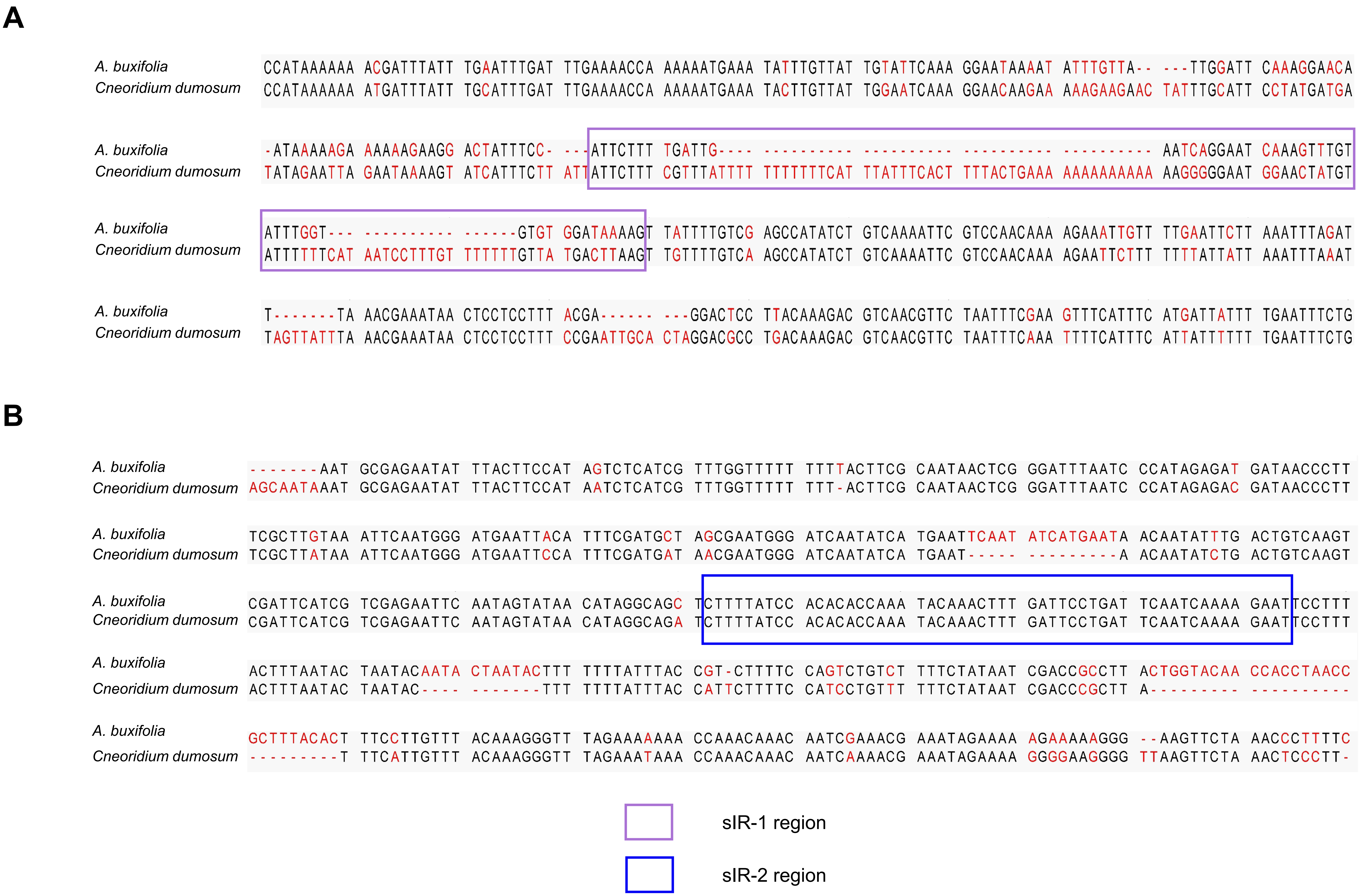
A
A. buxifolia CCATAAAAAA ACGATTTATT TGAATTTGAT TTGAAAACCA AAAAATGAAA TATTTGTTAT TGTATTCAAA GGAATAAAAT ATTTGTTA-- ---TTGGATT CAAAGGAACA
Cneoridium dumosum CCATAAAAAA ATGATTTATT TGCATTTGAT TTGAAAACCA AAAAATGAAA TACTTGTTAT TGGAATCAAA GGAACAAGAA AAAGAAGAAC TATTTGCATT CCTATGATGA
A. buxifolia -ATAAAAAGA AAAAAGAAGG ACTATTTCC- ---ATTCTTT TGATTG---- ---------- ---------- ---------- ---------- AATCAGGAAT CAAAGTTTGT
Cneoridium dumosum TATAGAATTA GAATAAAAGT ATCATTTCTT ATTATTCTTT CGTTTATTTT TTTTTTTCAT TTATTTCACT TTTACTGAAA AAAAAAAAAA AAGGGGGAAT GGAACTATGT
A. buxifolia ATTTGGT--- ---------- ------GTGT GGATAAAAGT TATTTTGTCG AGCCATATCT GTCAAAATTC GTCCAACAAA AGAAATTGTT TTGAATTCTT AAATTTAGAT
Cneoridium dumosum ATTTTTTCAT AATCCTTTGT TTTTTTGTTA TGACTTAAGT TGTTTTGTCA AGCCATATCT GTCAAAATTC GTCCAACAAA AGAATTCTTT TTTTATTATT AAATTTAAAT
A. buxifolia T-------TA AACGAAATAA CTCCTCCTTT ACGA------ ---GGACTCC TTACAAAGAC GTCAACGTTC TAATTTCGAA GTTTCATTTC ATGATTATTT TGAATTTCTG
Cneoridium dumosum TAGTTATTTA AACGAAATAA CTCCTCCTTT CCGAATTGCA CTAGGACGCC TGACAAAGAC GTCAACGTTC TAATTTCAAA TTTTCATTTC ATTATTTTTT TGAATTTCTG
B
A. buxifolia -------AAT GCGAGAATAT TTACTTCCAT AGTCTCATCG TTTGGTTTTT TTTTACTTCG CAATAACTCG GGATTTAATC CCATAGAGAT GATAACCCTT
Cneoridium dumosum AGCAATAAAT GCGAGAATAT TTACTTCCAT AATCTCATCG TTTGGTTTTT TTT-ACTTCG CAATAACTCG GGATTTAATC CCATAGAGAC GATAACCCTT
A. buxifolia TCGCTTGTAA ATTCAATGGG ATGAATTACA TTTCGATGCT AGCGAATGGG ATCAATATCA TGAATTCAAT ATCATGAATA ACAATATTTG ACTGTCAAGT
Cneoridium dumosum TCGCTTATAA ATTCAATGGG ATGAATTCCA TTTCGATGAT AACGAATGGG ATCAATATCA TGAAT----- ---------A ACAATATCTG ACTGTCAAGT
A. buxifolia CGATTCATCG TCGAGAATTC AATAGTATAA CATAGGCAGC TCTTTTATCC ACACACCAAA TACAAACTTT GATTCCTGAT TCAATCAAAA GAATTCCTTT
Cneoridium dumosum CGATTCATCG TCGAGAATTC AATAGTATAA CATAGGCAGA TCTTTTATCC ACACACCAAA TACAAACTTT GATTCCTGAT TCAATCAAAA GAATTCCTTT
A. buxifolia ACTTTAATAC TAATACAATA CTAATACTTT TTTTATTTAC CGT-CTTTTC CAGTCTGTCT TTTCTATAAT CGACCGCCTT ACTGGTACAA CCACCTAACC
Cneoridium dumosum ACTTTAATAC TAATAC---- -------TTT TTTTATTTAC CATTCTTTTC CATCCTGTTT TTTCTATAAT CGACCCGCTT A--------- ----------
A. buxifolia GCTTTACACT TTCCTTGTTT ACAAAGGGTT TAGAAAAAAA CCAAACAAAC AATCGAAACG AAATAGAAAA AGAAAAAGGG --AAGTTCTA AACCCTTTTC
Cneoridium dumosum ---------T TTCATTGTTT ACAAAGGGTT TAGAAATAAA CCAAACAAAC AATCAAAACG AAATAGAAAA GGGGAAGGGG TTAAGTTCTA AACTCCCTT-
sIR-1 region
sIR-2 region

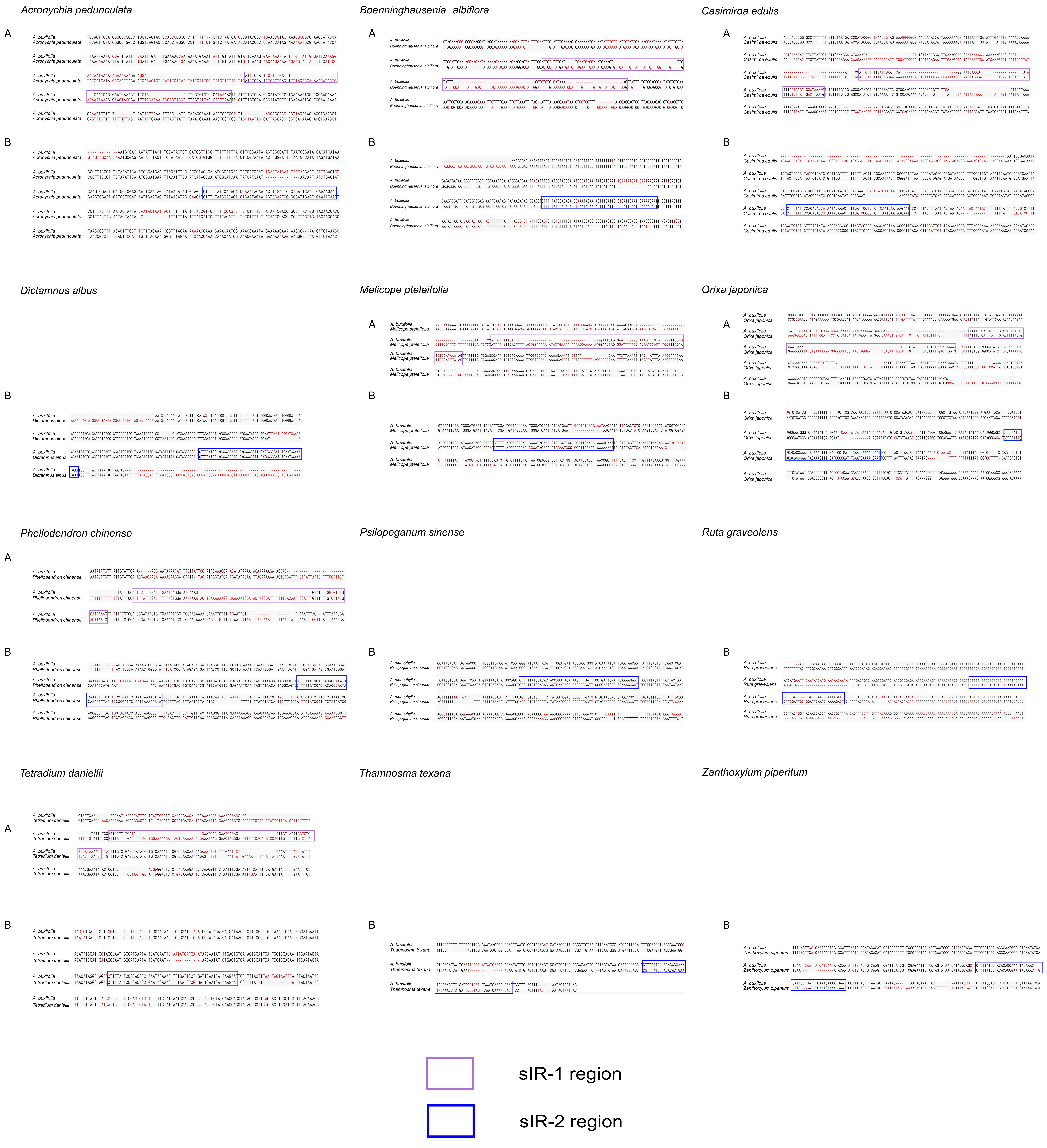

Acronychia pedunculata
Boenninghausenia albiflora
Casimiroa edulis
Dictamnus albus
Melicope pteleifolia
Orixa japonica
Phellodendron chinense
Psilopeganum sinense
Ruta graveolens
Tetradium daniellii
Thamnosma texana
Zanthoxylum piperitum
A
B
sIR-1 region
sIR-2 region

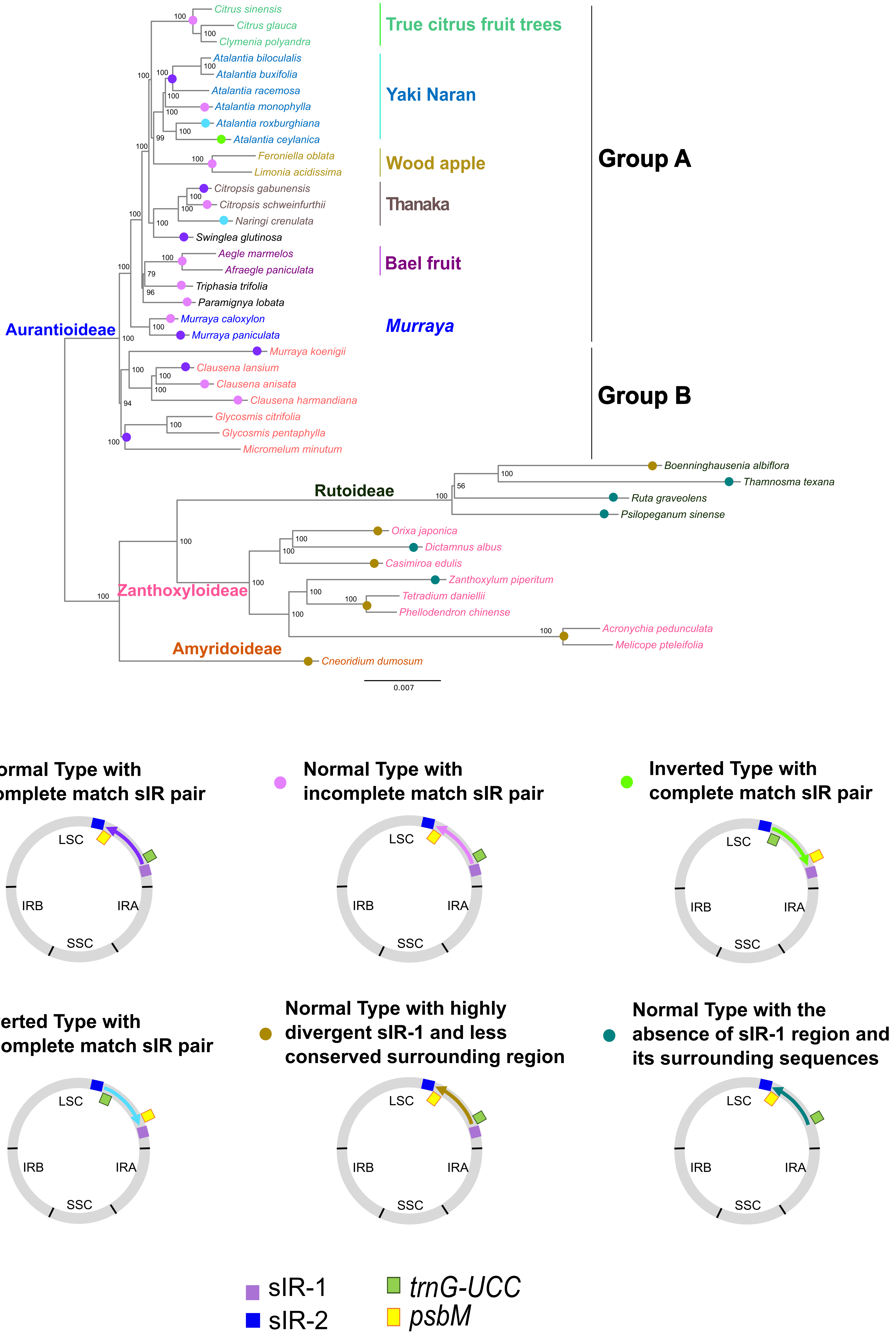
Citrus sinensis
Citrus glauca
Clymenia polyandra
True citrus fruit trees
Atalantia bilocularis
Atalantia buxifolia
Atalantia racemosa
Atalantia monophylla
Atalantia roxburghiana
Atalantia ceylanica
Yaki Naran
Feroniella oblata
Limonia acidissima
Wood apple
Citropsis gabunensis
Citropsis schweinfurthii
Naringi crenulata
Thanaka
Swinglea glutinosa
Aegle marmelos
Afraegle paniculata
Bael fruit
Triphasia trifolia
Paramignya lobata
Murraya caloxylon
Murraya paniculata
Murraya
Group A
Aurantioideae
Murraya koenigii
Clausena lansium
Clausena anisata
Clausena harmandiana
Glycosmis citrifolia
Glycosmis pentaphylla
Micromelum minutum
Group B
Boenninghausenia albiflora
Thamnosma texana
Rutoideae
Ruta graveolens
Psilopeganum sinense
Orixa japonica
Dictamnus albus
Casimiroa edulis
Zanthoxylum piperitum
Zanthoxyloideae
Tetradium daniellii
Phellodendron chinense
Acronychia pedunculata
Melicope pteleifolia
Amyridoideae
Cneoridium dumosum
0.007
Normal Type with complete match sIR pair
Normal Type with incomplete match sIR pair
Inverted Type with complete match sIR pair
Inverted Type with incomplete match sIR pair
Normal Type with highly divergent sIR-1 and less conserved surrounding region
Normal Type with the absence of sIR-1 region and its surrounding sequences
LSC
IRB
IRA
SSC
sIR-1
sIR-2
trnG-UCC
psbM

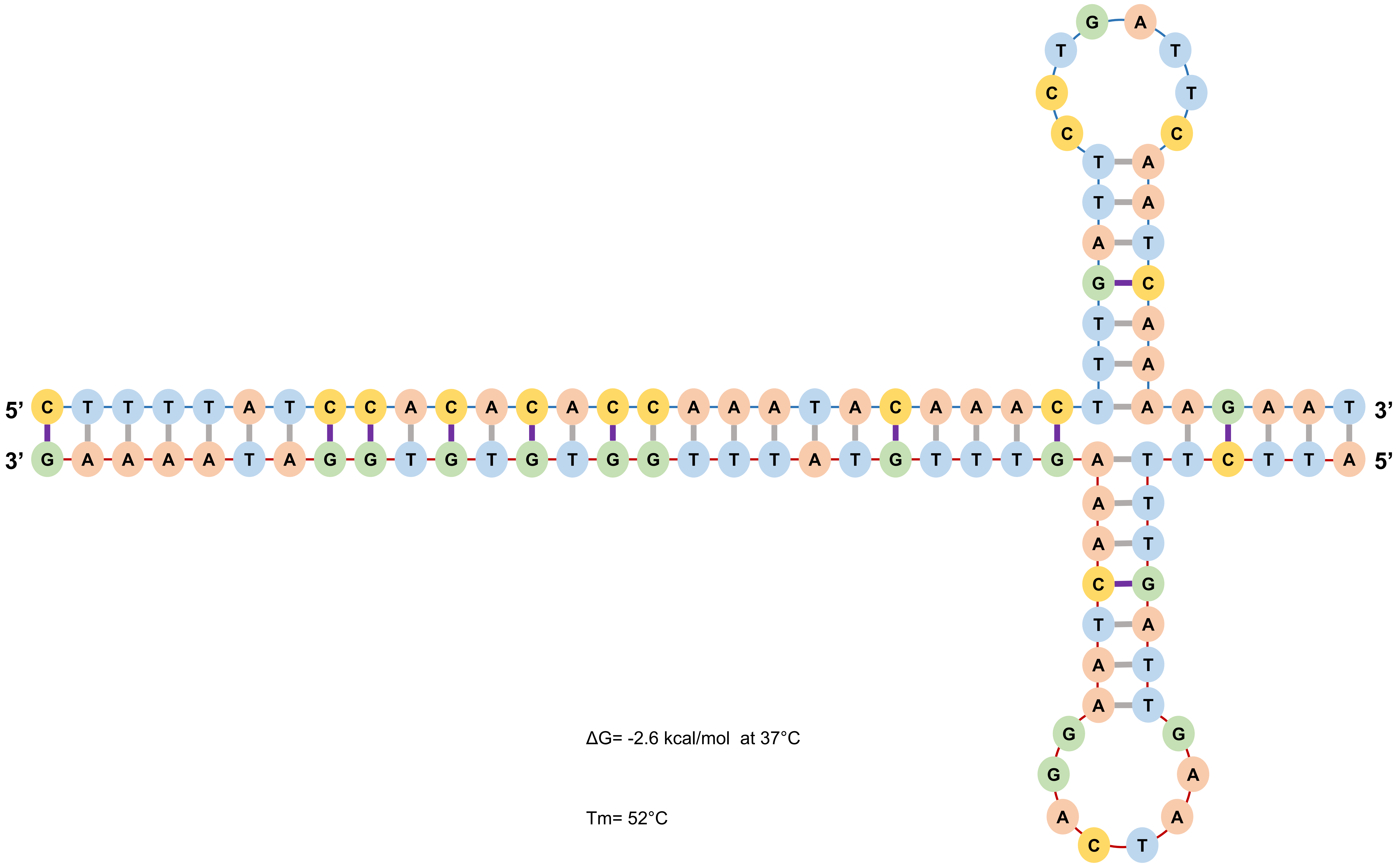
5' C T T T T A T C C A C A C A C C A A A T A C A A A C T A A G A A T 3'
3' G A A A A T A G G T G T G T G G T T T A T G T T T G A T T C T T A 5'
ΔG= -2.6 kcal/mol at 37°C
Tm= 52°C

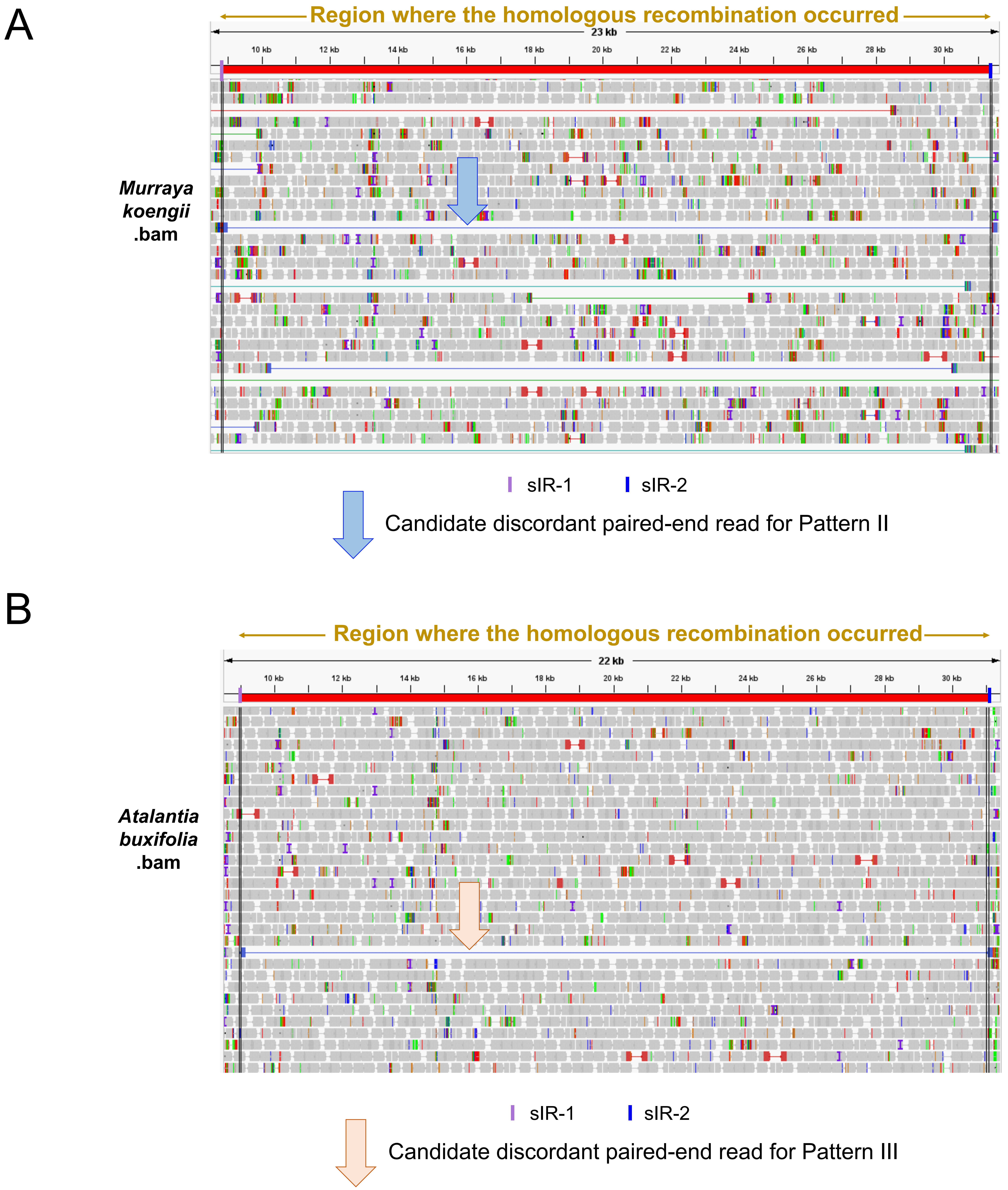
A
Region where the homologous recombination occurred
23 kb
10 kb
12 kb
14 kb
16 kb
18 kb
20 kb
22 kb
24 kb
26 kb
28 kb
30 kb
Murraya koengii .bam
sIR-1
sIR-2
Candidate discordant paired-end read for Pattern II
B
Region where the homologous recombination occurred
22 kb
10 kb
12 kb
14 kb
16 kb
18 kb
20 kb
22 kb
24 kb
26 kb
28 kb
30 kb
Atalantia buxifolia .bam
sIR-1
sIR-2
Candidate discordant paired-end read for Pattern III

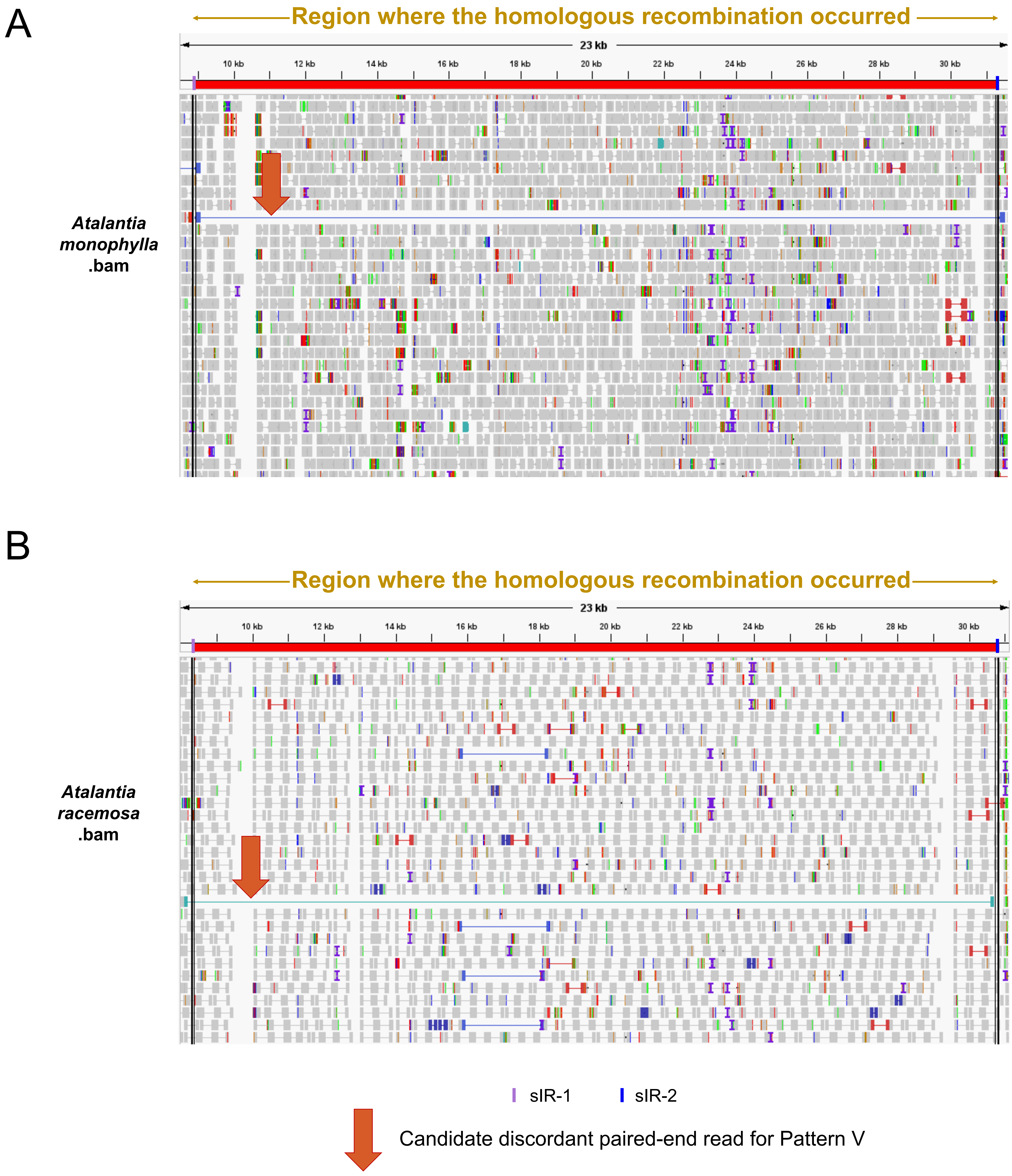
A
Region where the homologous recombination occurred
23 kb
10 kb
12 kb
14 kb
16 kb
18 kb
20 kb
22 kb
24 kb
26 kb
28 kb
30 kb
Atalantia monophylla .bam
B
Region where the homologous recombination occurred
23 kb
10 kb
12 kb
14 kb
16 kb
18 kb
20 kb
22 kb
24 kb
26 kb
28 kb
30 kb
Atalantia racemosa .bam
sIR-1
sIR-2
Candidate discordant paired-end read for Pattern V

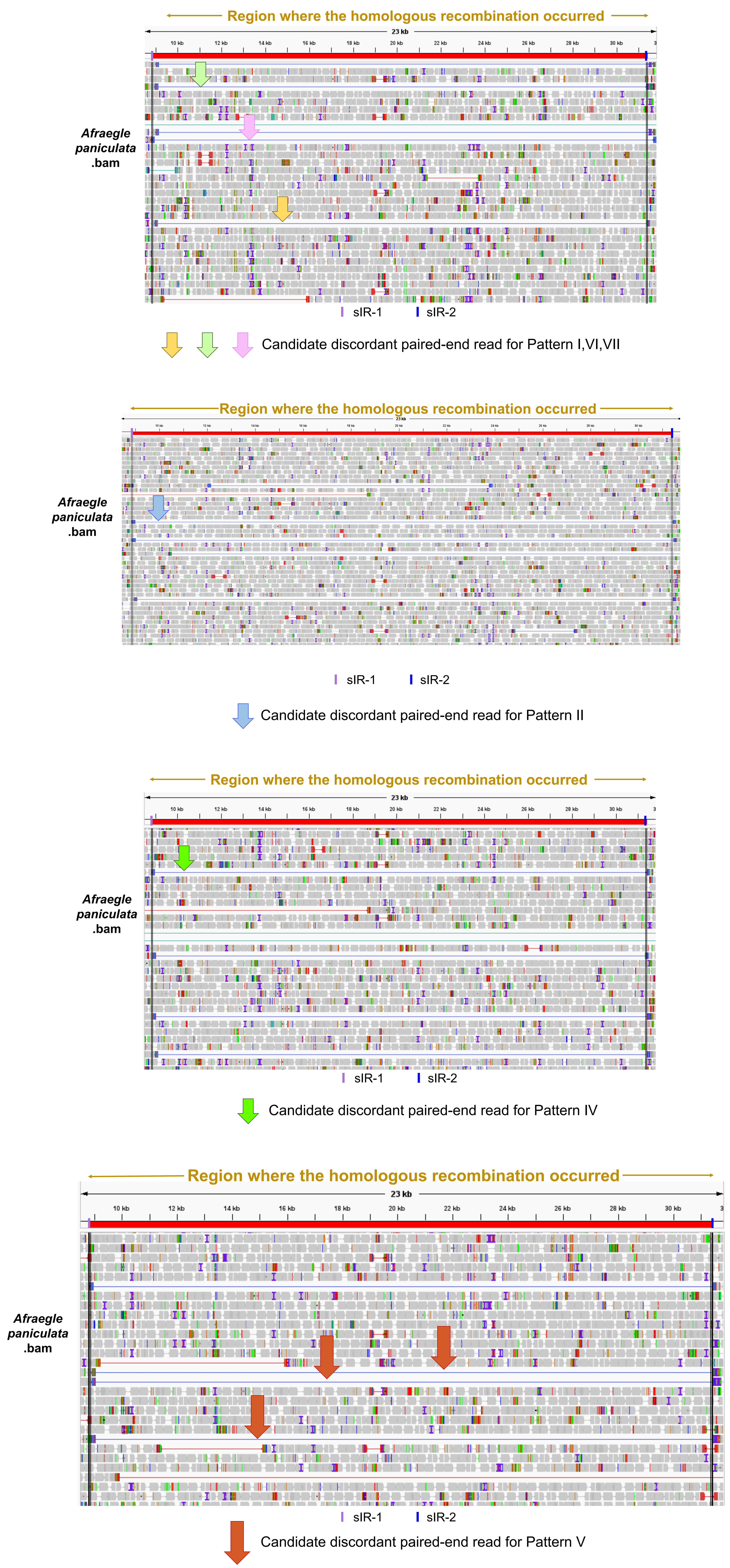
Region where the homologous recombination occurred
23 kb
10 kb
12 kb
14 kb
16 kb
18 kb
20 kb
22 kb
24 kb
26 kb
28 kb
30 kb
Afraegle paniculata .bam
sIR-1
sIR-2
Candidate discordant paired-end read for Pattern I,VI,VII
Region where the homologous recombination occurred
Afraegle paniculata .bam
sIR-1
sIR-2
Candidate discordant paired-end read for Pattern II
Region where the homologous recombination occurred
23 kb
10 kb
12 kb
14 kb
16 kb
18 kb
20 kb
22 kb
24 kb
26 kb
28 kb
30 kb
Afraegle paniculata .bam
sIR-1
sIR-2
Candidate discordant paired-end read for Pattern IV
Region where the homologous recombination occurred
23 kb
10 kb
12 kb
14 kb
16 kb
18 kb
20 kb
22 kb
24 kb
26 kb
28 kb
30 kb
Afraegle paniculata .bam
sIR-1
sIR-2
Candidate discordant paired-end read for Pattern V

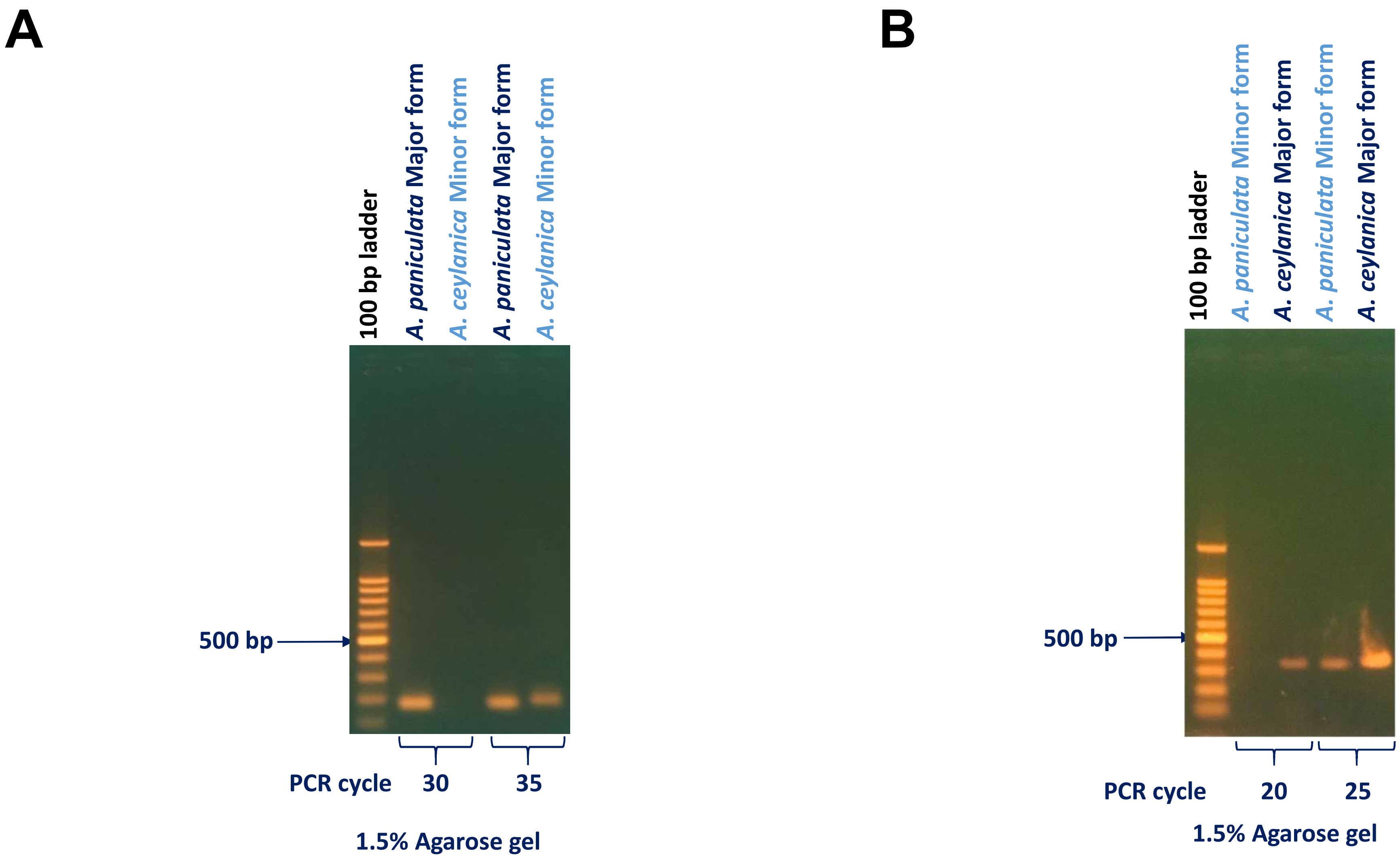
A
100 bp ladder
A. paniculata Major form
A. ceylanica Minor form
A. paniculata Major form
A. ceylanica Minor form
500 bp
PCR cycle
30
35
1.5% Agarose gel
B
100 bp ladder
A. paniculata Minor form
A. ceylanica Major form
A. paniculata Minor form
A. ceylanica Major form
500 bp
PCR cycle
20
25
1.5% Agarose gel

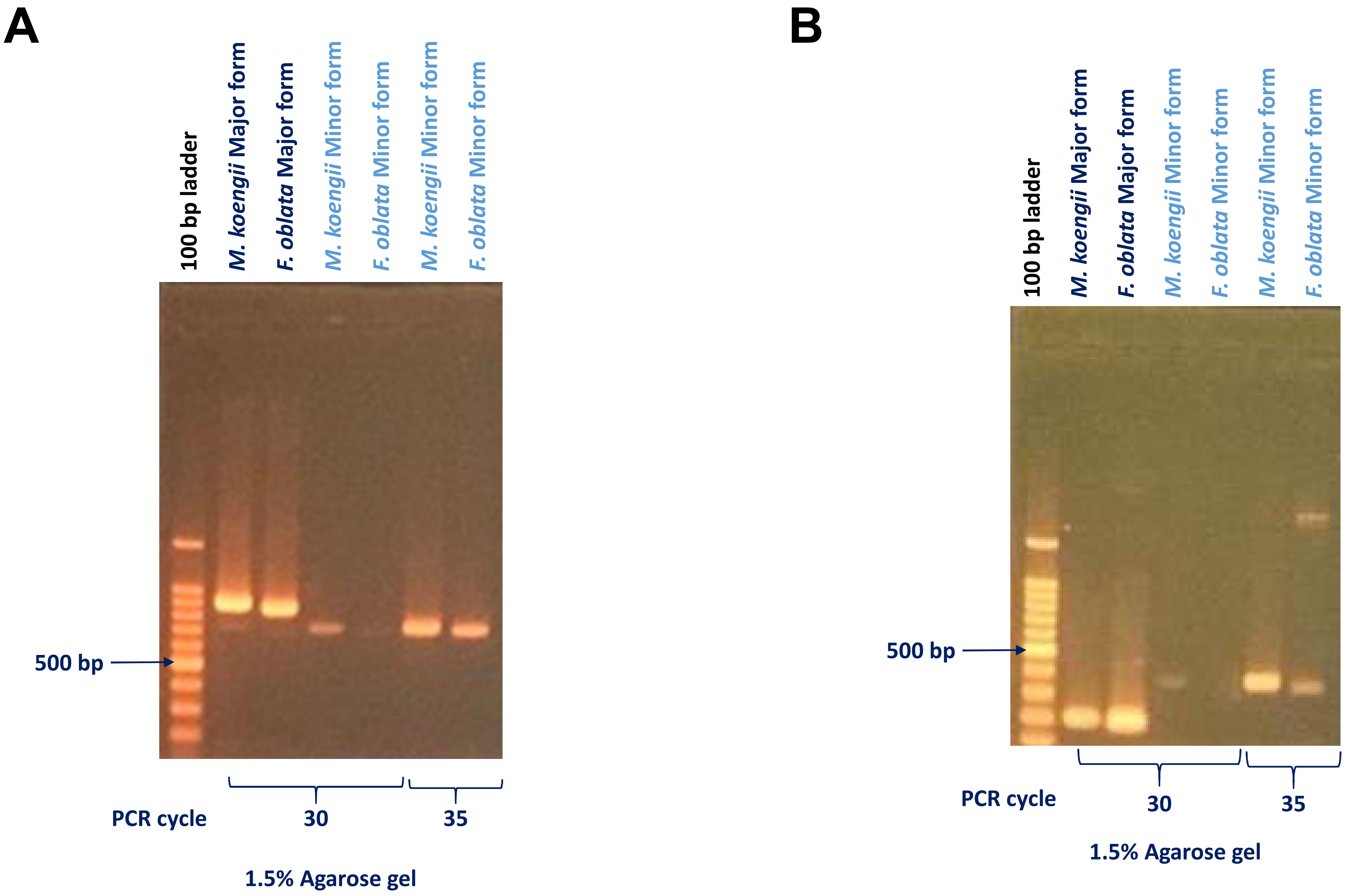

A
100 bp ladder
M. koengii Major form
F. oblata Major form
M. koengii Minor form
F. oblata Minor form
M. koengii Minor form
F. oblata Minor form
500 bp
PCR cycle
30
35
1.5% Agarose gel
B
100 bp ladder
M. koengii Major form
F. oblata Major form
M. koengii Minor form
F. oblata Minor form
M. koengii Minor form
F. oblata Minor form
500 bp
PCR cycle
30
35
1.5% Agarose gel

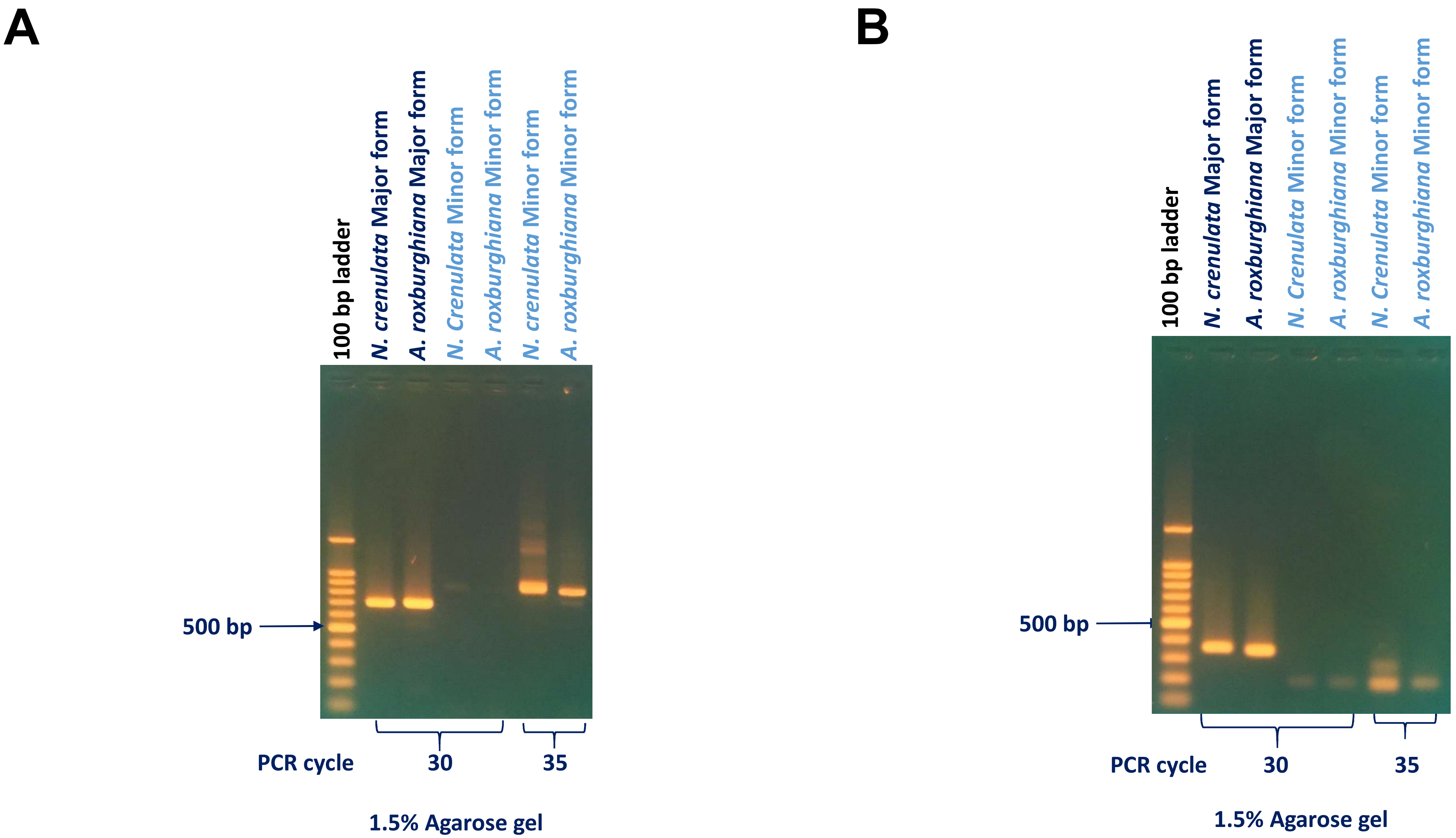

A
100 bp ladder
N. crenulata Major form
A. roxburghiana Major form
N. Crenulata Minor form
A. roxburghiana Minor form
N. crenulata Minor form
A. roxburghiana Minor form
500 bp
PCR cycle
30
35
1.5% Agarose gel
B
100 bp ladder
N. crenulata Major form
A. roxburghiana Major form
N. Crenulata Minor form
A. roxburghiana Minor form
N. Crenulata Minor form
A. roxburghiana Minor form
500 bp
PCR cycle
30
35
1.5% Agarose gel
